# Chronic obstructive pulmonary disease reprograms the lung into an immune organ through trained immunity, cell death networks, and immune checkpoint dysregulation

**DOI:** 10.3389/fmed.2026.1721780

**Published:** 2026-01-28

**Authors:** Fatma Saaoud, Keman Xu, Yifan Lu, Ying Shao, Baosheng Han, Xianwei Wang, Xiaohua Jiang, Xiaolei Liu, Jianhai Du, Hong Wang, Beata Kosmider, Xiaofeng Yang

**Affiliations:** 1 Lemole Center for Integrated Lymphatics and Vascular Research, Department of Cardiovascular Sciences, Lewis Katz School of Medicine, Temple University, Philadelphia, PA, United States; 2 Metabolic Disease Research and Thrombosis Research Center, Department of Cardiovascular Sciences, Lewis Katz School of Medicine, Temple University, Philadelphia, PA, United States; 3 Department of Ophthalmology and Visual Sciences, West Virginia University School of Medicine, Morgantown, WV, United States; 4 Center for Inflammation and Lung Research, Department of Microbiology, Immunology and Inflammation, Lewis Katz School of Medicine, Temple University, Philadelphia, PA, United States

**Keywords:** COPD, epithelial-to-mesenchymal transition (EMT), immune organs, secretomes, trained immunity

## Abstract

**Introduction:**

Chronic obstructive pulmonary disease (COPD) is a heterogeneous inflammatory disorder characterized by persistent immune dysregulation and progressive structural deterioration of the lung. However, how COPD reshapes lung architecture, immune signaling, and cellular identity at a systems level remains incompletely understood.

**Methods:**

We performed integrative, multi-dimensional transcriptomic analysis of human COPD lung datasets to evaluate alterations in immune signaling, regulated cell death pathways, fibrosis-associated programs, cell type–specific transcriptional identity, and immune checkpoint regulation. Genetic and cytokine-based perturbations targeting trained immunity pathways were analyzed to assess functional relevance.

**Results:**

COPD induced broad transcriptional activation of cytokines, secretory and plasma membrane proteins, CD markers, innate immune genes, and trained immunity genes. Deficiency of SET7, a promoter of trained immunity, or overexpression of IL-37, an inhibitor of trained immunity, attenuated expression of COPD-upregulated immune genes. COPD also promoted tissue injury through coordinated upregulation of genes regulating multiple forms of regulated cell death, including autosis, autophagy, parthanatos, immunogenic cell death, mitochondrial permeability transition-associated death, lysosomal cell death, apoptosis, necroptosis, ferroptosis, mitotic cell death, and proliferation-associated cell death. In parallel, COPD enhanced epithelial-to-mesenchymal transition and fibrosis-related transcriptional programs. Transcriptomic identity was disrupted in 10 of 14 major human lung cell types, with evidence of pathological trans-differentiation marked by aberrant expression of over 50 cell type-specific marker genes. Alveolar macrophages exhibited extensive dysregulation of immune checkpoint ligand; notably, PVR (CD155) expression was reduced in severe emphysema, while experimental PVR overexpression suppressed pro-inflammatory gene expression in both alveolar and interstitial macrophages. Additionally, COPD impaired the suppressive capacity of CD4^+^Foxp3^+^ regulatory T cells through downregulation of key immunosuppressive genes, including those associated with FoxP3^+^, TIGIT^+^, and LPS-responsive Tregs. Shared immunosuppressive gene signatures were identified between PVR-overexpression-inducing CD4^+^ T cells and IL-10-mediated regulatory pathways in T cells and monocytes.

**Discussion:**

Collectively, these findings demonstrate that COPD reprograms the lung toward an immune-like organ by promoting immune cell-like trans-differentiation of structural cells, activating diverse regulated cell death pathways, and altering immune checkpoint signaling. These mechanisms highlight potential therapeutic targets for immunomodulatory intervention in COPD.

## 1 Introduction

Chronic obstructive pulmonary disease (COPD) is a major public health burden and a leading cause of morbidity and mortality in the United States, accounting for approximately 15.4 million physician visits, 1.5 million emergency department visits, and 726,000 hospitalizations annually (1). Globally, COPD affects ∼ 10% of adults over 40 years and remains the third leading cause of death worldwide (2, 3). COPD is a progressive and heterogeneous disease characterized by chronic airway inflammation, airflow obstruction, and alveolar destruction (emphysema), ultimately resulting in irreversible loss of lung function (4). Impaired host defense mechanisms increase susceptibility to pathogens, pollutants, toxic chemicals, and allergens, establishing a dynamic interplay between environmental exposures and immune responses. These exposures perturb the lung microbiome and disrupt immunoregulatory pathways, contributing to chronic inflammation and disease progression. COPD encompasses multiple clinical phenotypes that are thought to rises from distinct molecular endotypes (5).

Beyond the lung, chronic pulmonary inflammation leads to systemic spillover of cytokines, chemokines, and other inflammatory mediators (6), promoting low-grade systemic inflammation and a broad spectrum of comorbidities, including cardiovascular disease (7), pulmonary vascular disease, venous thromboembolism, metabolic dysfunction, diabetes, lung cancer, neuropsychiatric disorders, osteoporosis, anemia, and cognitive impairment (8). We recently reported that up to 53% of human proteins can be secreted during inflammation through six distinct pathways, including canonical, caspase-1-dependent, caspase-4/11-dependent, exosome-mediated (9), Weibel-Palade body-mediated, and autophagy-mediated release (10).

Epithelial–mesenchymal transition (EMT) is a key pathological process in COPD, where airway epithelial cells progressively lose their epithelial characteristics and acquire mesenchymal features, including increased vimentin and α-SMA expression. This process, heavily triggered by cigarette smoke, contributes to airway remodeling, fibrosis, inflammation, and tissue destruction through the generation of fibrotic stromal cells. Several signaling pathways—particularly Wnt family member (Wnt)/β-catenin, transforming growth factor-β (TGF-β), and phosphoinositide 3-kinase (PI3K)/protein kinase B (AKT)—are known to regulate this transition (11–15). However, the full spectrum of transcriptomic alterations associated with innate immune responses (16), upregulation of cytokines (17) and secretomes (10, 18), trained immunity (19–21), regulated cell death pathways (22, 23), and cell trans-differentiation (24), including epithelial-to-mesenchymal transition (EMT) and fibrosis remains incompletely defined.

The respiratory tract is constantly exposed to inhaled pathogens and toxic substances. Antigen-presenting cells (APCs) orchestrate innate and adaptive immune responses by processing and presenting antigenic peptides to T cells (25). Cigarette smoke reduces CD4^+^Foxp3^+^ regulatory T cells (Treg) populations, with significantly lower Treg frequencies are significantly lower, observed in smokers compared with non-smokers (26). Tregs play a central role in restraining pulmonary inflammation in COPD (27). We previously demonstrated that chronic exposure to cigarette smoke combined with morphine further decreases CD4^+^ Tregs through transcriptomic reprogramming (28) and promotes Treg plasticity toward a pro-inflammatory CD4^+^ T helper cell 17 (Th17) phenotype (29), in part via trained immunity mechanisms (30). Cigarette smoke also promotes inflammation through reactive oxygen species (ROS)-driven induction of trained immunity and trained tolerance mechanisms (31). Professional APCs—monocytes, macrophages, and dendritic cells—present antigenic peptides via major histocompatibility complex (MHC) molecules to CD4^+^ and CD8^+^ T cells (32, 33). Moreover, a range of non-professional APCs, including alveolar epithelial cells, endothelial cells (16, 34, 35), vascular smooth muscle cells (36), fibroblasts, innate lymphoid cells (ILCs), eosinophils, mast cells, and other interstitial cell populations, can express MHC class II and modulate CD4^+^ T cell activation and differentiation (25).

Given the centrality of T cell–APC interactions in immune regulation (37), immune checkpoint inhibitors have emerged as critical modulators and form the basis of modern cancer immunotherapies (37, 38). COPD is the most common comorbidity in lung cancer patients (39), and as immunotherapy extends survival, it becomes increasingly important to understand immune checkpoint expression and signaling in COPD. However, despite their relevance, immune checkpoint dysregulation in COPD remains poorly defined.

Taken together, several major gaps remain in our understanding of COPD immunopathology. Key unanswered questions include whether COPD induces transcriptomic reprogramming across the full spectrum of cytokines, chemokines, secretomes, plasma membrane proteins, CD markers, innate immune genes, and trained immunity; whether COPD alters regulators spanning all 14 known forms of regulated cell death; and whether master regulators of tissue remodeling—particularly EMT, fibrosis, and pathological trans-differentiation—are dysregulated. To address these gaps, we applied a novel, knowledge-based, multi-dimensional transcriptomic analysis platform to systematically interrogate molecular and cellular reprogramming in COPD. Here, we provide a comprehensive characterization of COPD-associated transcriptional dysregulation and uncover new mechanisms by which COPD transforms the lung into an immune organ.

## 2 Materials and methods

### 2.1 Transcriptomic data collection

To investigate gene expression changes and immunoregulatory pathways in COPD, we curated and analyzed publicly available transcriptomic datasets from the NIH-NCBI Gene Expression Omnibus (GEO) database.^1^ These datasets encompassed both human and mouse studies across multiple tissue types and experimental conditions relevant to lung inflammation, immune regulation, and COPD pathogenesis. The following datasets included: COPD lung tissue: whole-lung transcriptome from 11 COPD patients and 11 healthy controls (GSE239897); SET domain containing 7, histone lysine methyltransferase (SET7) knockout: H9 human embryonic stem cells transfected with SET7 siRNA (*n* = 2; GSE53038); Interleukin-37 (IL-37) overexpression: white adipose tissue from wild-type and human IL-37-overexpressing transgenic mice on a high-fat diet (GSE58952); alveolar macrophages from normal non-smokers (*n* = 24), normal smokers (*n* = 34), and smokers with COPD (*n* = 12) (GSE13896); poliovirus receptor (PVR) overexpression: CD4^+^ T cells stimulated with PVR-overexpressing L cells (*n* = 4, GSE194293); severe emphysema lung tissue: lungs from smokers with severe emphysema (*n* = 18) and mild/no emphysema (*n* = 12) (GSE1650); lipopolysaccharide (LPS)-stimulated macrophages: monocytes-derived macrophages treated with LPS (*n* = 3; GSE5099); FoxP3^+^ Tregs (*n* = 3; GSE164460); LPS-treated resolving lung Tregs (*n* = 3; GSE104088); T cell immunoreceptor with Ig and ITIM domains (TIGIT)^+^ Tregs (*n* = 2; GSE56299); IL-10 (an anti-inflammatory/immunosuppressive cytokine)-treated CD4^+^ T cells (*n* = 3; GSE17199 and GSE198963); IL-10–treated monocytes: peripheral blood mononuclear cells (PBMC)-derived monocytes treated with IL-10 (*n* = 3; GSE43700); LPS and IL-10 blocking antibody treatment: human monocyte-derived macrophages stimulated with LPS and IL-10 blocking antibody (*n* = 3; GSE181250); IL-10–stimulated monocyte-derived DCs (*n* = 3; GSE180761). All datasets were downloaded from the publicly accessible NIH-NCBI GEO portal (see text footnote 1). Each dataset was curated and pre-processed for downstream analysis. Differential gene expression analysis was conducted using the GEO2R tool,^2^ allowing statistical comparisons between experimental and control groups within each dataset. Genes with *p*-value < 0.05 and log_2_ fold change (log_2_FC) > 1 or < -1 were considered significantly differentially expressed.

### 2.2 Metascape pathway analysis

To assess the biological relevance of the differentially expressed genes, pathway enrichment analysis was conducted using Metascape^3^ as we reported (36, 40). Gene lists derived from transcriptomic comparisons were uploaded to Metascape for annotation and enrichment analysis across multiple curated databases, including Gene Ontology (GO), KEGG, and Reactome. An enrichment threshold of adjusted *p*-value < 0.01 was applied. The Metascape outputs were used to identify enriched biological processes, molecular functions, and signaling pathways related to immune regulation, cell death, fibrosis, EMT, and inflammation.

## 3 Results

### 3.1 COPD-induced inflammatory and innate immunity pathways

**FIGURE 1:**
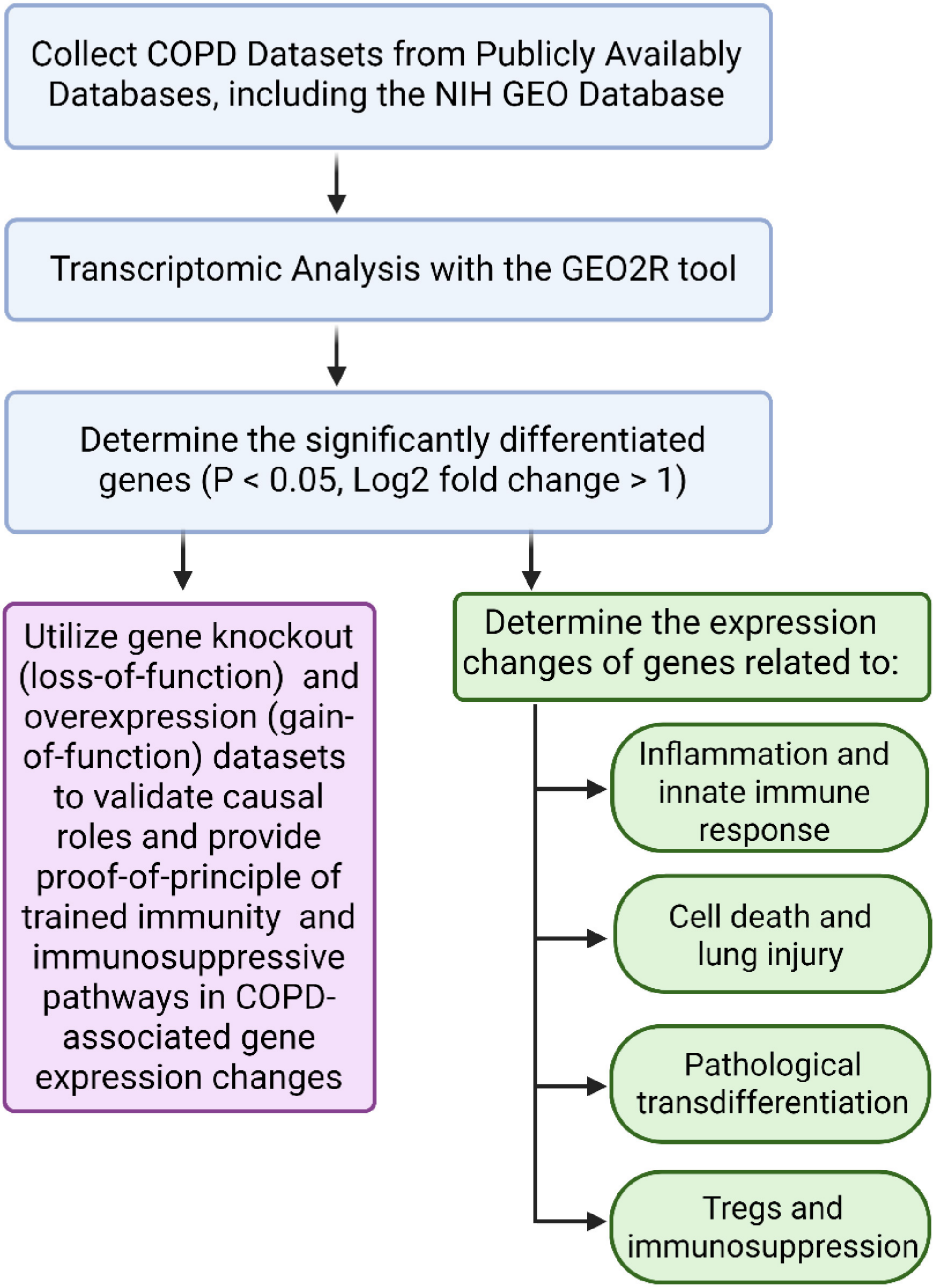
Workflow diagram of the study. Chronic Obstructive Pulmonary Disease (COPD) transcriptomic datasets were obtained from the National Institutes of Health (NIH). Differential gene expression analysis was performed using GEO2R, identifying significantly altered genes with a log_2_ fold change (log_2_FC) > 1 and *P* < 0.05. Subsequent analyses focused on genes associated with inflammation, immune response, pathological transdifferentiation, cell death, and lung injury. Additionally, loss-of-function and gain-of-function datasets were utilized to validate key findings and provide proof-of-concept for the involvement of trained immunity and immunosuppressive pathways in COPD progression.

Cytokines are established mediators of chronic inflammation in COPD (2). We hypothesized that in COPD, the secretory proteins—including cytokines, chemokines, and secretomes (41)—may drive systemic inflammation and comorbidities. To investigate how COPD alters inflammatory and innate immune pathways, we analyzed bulk RNA-Seq dataset from lung tissue of COPD patients available in the NIH-NCBI-GEO database (GSE239897) (4, 42). We applied a knowledge-based transcriptomic profiling approach previously developed by our group (17, 19, 40) (Figure 1).

As summarized in Table 1, COPD lungs demonstrated significant upregulation in the expression of 49 of 1,325 cytokine/chemokine genes (3.7%), 339 of 9,525 secretome genes (3.6%), 45 of 2,202 plasma membrane protein-encoding genes (2.04%), 24 of 384 CD marker genes (6.3%), 39 of 1,615 innate immune genes (2.4%), and six of 101 trained immunity genes (5.9%).

**TABLE 1:** Transcriptomic profiling identifies COPD-associated inflammatory, innate immune, and trained immunity gene signatures.

Gene list/source	Gene number and percentage	COPD upregulated genes (Log2FC > 1, *P* < 0.05), (GSE239897)
Cytokine/chemokine genes (HPA)	49/1325 (3.77%)	CXCL13 SELE ALOX15 CXCL6 PTGS2 NR4A3 EGR1 SOCS2 IL6 CCL22 CD24 CSF3 LRCH1 ZFP36 VCAM1 SOX2 CCR7 FOS EFHC2 IRF4 CCL5 PLD4 JUNB CCL11 GPR15 CCL19 LIF TRIL FAM3D ABCD2 CCL3 OSM CCL4 CXCL3 CCR4 CXCL12 FOSL1 CFAP99 DUSP1 CXCL2 LTF AREG DCDC1 SCGB3A1 IL5RA ZC3H12A SEPTIN1 FOXC1 CD96
Secretomes	339/9525 (3.6%)	ABO ADAM28 ADAMTS4 ADGRE3 AGR2 AGR3 AMY1B ANOS1 APLN ART4 ASPN AZGP1 B3GNT3 BMP2 BMP3 BMP4 BMP5 BMP7 BPIFB1 C1QTNF7 C4A C4B C8B CASQ2 CCDC3 CCL11 CCL19 CCL2 CCL22 CCL3 CCL4 CCL5 CCNL1 CD1E CD200R1 CD24 CD40LG CD6 CD8A CD8B CDHR4 CEACAM5 CFAP65 CFD CHIT1 CHL1 CHST6 CHST9 CKMT1B CLCF1 CNTNAP3 COL28A1 COL6A6 CORIN CP CPA3 CPE CPXM2 CRLF2 CSF3 CTHRC1 CTSV CX3CL1 CXCL1 CXCL12 CXCL13 CXCL14 CXCL2 CXCL3 CXCL5 CXCL6 CXCL8 CXCL9 CYP21A2 CYP2C18 CYP2C19 CYP2C9 DEPP1 DHRS9 DKK2 DNASE1L3 DNASE2B DPT ECM2 ENPP2 ENPP3 EPHA3 ERAP2 EREG FAM3D FASLG FBN3 FCMR FCRL5 FGFBP1 FGFR3 FGG FOLR2 FRAS1 FREM1 FRZB FTCDNL1 GALNT6 GOLM1 GZMK GZMM HBEGF HRC HTRA3 ICOS IGLL5 IHH IL1B IL1RN IL5RA IL6 INHBA INHBB ITGBL1 JCHAIN KDM6A LAG3 LIF LRFN5 LRRC32 LTBP2 LTF LUM MASP1 MFAP2 MFAP4 MMP19 MSMB MUC15 MUC16 MUC20 MUC4 MUC5B MZB1 NOTUM NRG4 NTN1 OSM PAMR1 PAPPA2 PDGFD PDZD2 PLA2G12B PLA2G7 PODNL1 PRSS12 PTGDS PTGS2 PZP RARRES1 RARRES2 SCGB3A1 SEMA3D SEMA3E SEMA6D SERPIND1 SERPINE1 SFRP2 SFRP5 SLAMF1 SMOC2 SNCA ST8SIA6 STOX1 SULF1 TFF3 TFPI2 THBS1 THBS2 TNF TNR TPSAB1 TPSB2 TRABD2A TUB TXNDC5 VEGFD VMO1 VWA3A VWA7 WFDC2 WIF1 XCL2 LDLR ACTN2 AREG HBD LMNA HBB HBG1 ABCD2 WEE1 SIT1 WDR49 SPAG1 CD3D RPGRIP1L CFAP45 SH2D1A TEKT3 MAPK8IP1 MYH10 HOMER1 JMJD1C CLDN1 CFAP58 CCR4 RGS6 MAP1A PHLDB2 HLA-DQA1 DNAH7 ALDH1A1 HSPA4L CD79B ITK TENM3 SYTL2 CAPS2 CFAP43 HBG2 NAT1 TSPAN1 KLHL41 ATP6V0D2 MYADM MS4A1 STEAP4 GJB1 KIF21A PFN1 SLC19A2 CD2 SERPINB3 LY9 IRF6 KRT5 TTC16 SLC28A3 LYPD3 IQCG THEMIS ITGB8 SLC4A8 CXCR4 RHPN2 GFRA1 KRT16P2 PGM2L1 S100P SPEF2 IGLC1 HBA2 C16orf54 TSPAN6 FBXO15 TRPV4 SLCO1A2 KDM6B SLA2 KIF6 SLC44A4 CLDN3 TNFRSF21 ARMC3 ANO1 GSTM5 CD3G RBP5 GJA1 FUZ SHISA2 VWA3B PROM1 CD3E ABCB1 CD5 DLG2 MYCBPAP TRAT1 CD74 TMPRSS4 TTC30A ROBO2 CAMK4 NFKBIZ SLAMF6 NEK10 CTNND2 SLC4A11 ALDH3A1 GSTA2 DNAH5 STAG3 LGR4 FOLH1 FERMT1 USP2 ARHGAP15 DUSP26 CD1C RGS16 FHAD1 PELI1 CD38 EPHA2 HLA-A PRKAA2 TEKT1 SLC7A2 EFHC1 PLCB4 TACSTD2 DNAAF1 ATPAF2 ADD3 LDHAL6B BTG2 MOXD1 TUBA4B THY1 RAB37 ITM2C MAK ATP6V1B1 DAPP1 DNAH12 SKAP1 STOML3 CFAP70 MAP3K19 CNN1 GSTA1 SLC27A2
Plasma membrane protein-encoding genes (HPA)	45/2202 (2.04%)	SERPINB3 MS4A1 S100A2 TRIB1 LY9 ZNF474 VCAM1 SOCS3 STOML3 EFHC2 USP2 RRAD CFAP206 CEACAM5 CNGA4 CFAP251 CFAP65 CCDC170 SLCO1A2 GAS2 LAX1 FLACC1 CTSV MUC20 TRAT1 DNAAF1 KLHL41 CCR4 SLC4A8 KIF19 CORIN ZMYND10 CFAP299 CFAP58 HYDIN CD3E LRRIQ3 PDZD2 ANKFN1 CD8B TEKT3 DAW1 SYTL2 CFAP157 SLC16A9
Cluster of differentiation (CD) marker genes (HPA)	24/384 (6.25%)	SELE CD79A PROM1 CD69 FCRL5 CD24 MS4A1 TNFRSF17 LY9 CD27 CD207 VCAM1 CCR7 CD5 CEACAM5 CD1C CD1E SLAMF7 CCR4 BMPR1B CD3E CD8B IL5RA CD96
Innate immune genes (Innatome database)	39/1615 (2.41%)	CXCL13 SELE IGLL5 PTGS2 NR4A3 TP63 BPIFB1 EGR1 SOCS2 IL6 ATF3 FCRL5 NR4A1 LY9 CD27 ZFP36 SOCS3 CCR7 FOS IRF4 CCL5 USP2 KLB TRIL UBD FCER1A NFKBIZ TRAT1 SERPINE1 OSM SLAMF7 CCR4 CXCL12 HBEGF DUSP1 CXCL2 NLRP14 MS4A2 ZC3H12A
Trained immunity genes (trained immunity database, TIDB)	6/101 (5.94%)	SELE IL6 CD207 CCL3 DUSP1 LTF
SET7 knockout downregulated genes (GSE53038, log2FC > 1, *P* < 0.05)	58/426 (COPD upregulated genes) (13.61%)	HBB CXCL13 HMGCS2 KRT17 MSMB CD79A CXCL6 PTGS2 SOCS2 PAMR1 IL6 KRT23 CHST6 S100A2 HS3ST2 CAPSL C7orf57 DNER VWA3B LOC100505841 VCAM1 CCR7 CCL5 RRAD SPATA18 ITGBL1 TTC29 DUSP5 LIF TTC16 GADD45B PAPPA2 FRZB ADAMTS4 CYP2C19 NEK10 DUSP26 FCER1A CCL3 KRT16P2 MIR205HG IER3 SERPINE1 CCL4 ABO SLC22A4 HBEGF FOSL1 CD3E NNMT FOXE1 AREG MS4A2 SYTL2 ZC3H12A VWA3A CAPS2 S100P
Trained immunity inhibitor (IL-37) overexpression downregulated genes (GSE58952, log2FC > 1, *P* < 0.05)	12/426(COPD upregulated genes) (2.81%)	DHRS9 ATF3 EGR2 STAP1 CDHR3 DNAH6 UBD CCL3 SPEF2 RGS1 ATP6V0D2 KCNJ16

Differential expression analysis revealed that COPD significantly upregulated genes involved in key immunological processes, including cytokine and chemokine signaling, secreted proteins, plasma membrane proteins, cluster of differentiation (CD) markers, innate immune pathways, and trained immunity programs. Among the 426 COPD-upregulated genes, deletion of the trained-immunity–promoting enzyme SET7 reduced the expression of 58 genes, whereas overexpression of the trained-immunity inhibitor IL-37 downregulated 12 genes, indicating that trained immunity pathways contribute to COPD-related transcriptional reprogramming. All transcriptomic data were obtained from the NIH-NCBI Gene Expression Omnibus (GEO) database: the COPD dataset (GSE239897), SET7 knockout dataset (GSE53038), and the IL-37 overexpression dataset (GSE53038). Differential expressed genes were defined using threshold of log*2* fold change (log*2*FC) > 1 and *P* < 0.05. Gene sets were downloaded from the Human Protein Atlas database (HPA, https://www.proteinatlas.org/), and innate immune gene list were cured from InnateDB (https://www.innatedb.com/).

To explore the contribution of trained immunity, we assessed gene suppression SET7 (a promoter of trained immunity) knockout (SET7-KO) and IL-37 (an inhibitor of trained immunity) overexpression model—both known to interfere with trained immunity (43, 44). SET7-KO downregulated 58 of 426 COPD-upregulated genes (13.6%), while IL-37 overexpression suppressed 12 of these genes (2.8%). These finding suggest that trained immunity plays a significant role in COPD pathogenesis, consistent with earlier studies highlighting innate immunity’s role in COPD (45). Metascape pathway enrichment analysis identified significantly enriched pathways linked to each group of COPD-upregulated inflammatory and innate immune genes (Supplementary Table 1), with most pathways unique to specific gene sets—highlighting functional diversity. Collectively, the upregulation of cytokine, chemokine and secretome genes likely represent a central mechanism by which COPD promotes lung and bronchial inflammation, systemic inflammation and multiple comorbidities.

### 3.2 COPD-induced lung injury is associated with upregulation of genes involved in regulated cell death

It is well established that four forms of regulated cell death—apoptosis (46), necroptosis (47), pyroptosis (inflammatory cell death) (48), and ferroptosis (49)—contribute to the pathogenesis of COPD. In 2018, the International Nomenclature Committee on cell death classified 12 distinct forms of regulated cell death, including intrinsic and extrinsic apoptosis, mitochondrial permeability transition (MPT) cell death, necroptosis, ferroptosis, pyroptosis, parthanatos, entotic cell death, neutrophil extracellular traps (NET)otic cell death, lysosome-dependent cell death (LDCD), autophagy-dependent cell death (ADCD), and immunogenic cell death (ICD) (50). We hypothesized that COPD may contribute to lung tissue injury by broadly upregulating genes associated with multiple regulated cell death pathways.

Transcriptomic analysis revealed that COPD significantly upregulated genes linked to all 12 forms of regulated cell death (Table 2). Specifically, we identified differential expression of 21 of 581 pyroptosis genes (3.6%), four of 346 apoptosis genes (1.2%), three of 239 necroptosis genes (1.3%), eight of 494 ferroptosis genes (1.6%), 35 of 1,831 autosis genes (1.9%), six of 359 autophagy genes (1.7%), four of 301 parthanatos genes (1.3%), 62 of 3,452 immunogenic cell death genes (1.8%), four of 204 MPT genes (2.0%), three of 233 lysosomal cell death genes (1.3%), 21 of 1,701 mitotic cell death genes (1.2%), and 31 of 1,782 proliferation-associated cell death genes (1.7%).

**TABLE 2:** COPD promotes diverse forms of programmed cell death.

Cell death type	The number and percentage of upregulated genes	COPD Upregulated cell death genes (log2FC > 1, *P* < 0,05), (GSE239897)
Pyroptosis	**21**/581 (3.61%)	CXCL13 GSTA2 POU2AF1 EGR1 CD69 CHL1 IL6 EGR2 VCAM1 IRF4 CCL19 PFN1 DUSP5 CCN2 FRZB SLC18A2 HBEGF ROPN1L CXCL2 NNMT HLA-DQA1
Apoptosis	**4**/346 (1.16%)	PTGS2 GAS2 ABO BMPR1B
Necroptosis	**3**/239 (1.26%)	FOS PFN1 GAS2
Ferroptosis	**8**/494 (1.62%)	FAM30A MS4A1 CLDN10 NME5 LINC00312 CP WEE1 MS4A2
Autosis	**35**/1831 (1.91%)	PTGS2 CCN1 EGR1 IL6 TSPAN1 ATF3 TRIB1 ZFP36 VCAM1 FOS IRF4 RRAD DUSP5 LIF KLF10 CCN2 IER3 SERPINE1 FERMT1 FGFBP1 MLF1 CXCL3 BHLHE40 SLC18A2 HBEGF DUSP1 CXCL2 NNMT JUND HLA-DQA1 DYRK3 PDZD2 AREG TSC22D2 ZC3H12A
Autophagy	**6**/359 (1.67%)	IL6 IRF4 ATP6V1B1 PIM2 ZC3H12A ATP6V0D2
Parthanatos	**4**/301 (1.33%)	TP63 BTG2 EYA1 CXCL12
Immunogenic cell death	**62**/3452 (1.8%)	CXCL13 IGLL5 IGLC1 FOSB CD79A CXCL6 MUC16 PTGS2 MZB1 SOCS2 CD69 IL6 ATF3 KRT23 EGR2 MORN5 NR4A1 EYA4 LY9 CD27 CFAP126 VCAM1 SOCS3 FOS IRF4 BTG2 RRAD HAS2 C6orf118 LIF LRRC46 GADD45B KLF10 SPEF1 CCN2 NFKBIZ IER3 SERPINE1 BHLHE40 ATXN7 SEMA3E HBEGF FOSL1 RHOH BMPR1B REM1 DUSP1 CABCOCO1 PACRG CD3E CCDC103 NNMT DYRK3 CD8B AREG SCGB3A1 IL5RA TSC22D2 LMNTD1 SMOC2 RSPH9 FOXC1
Mitochondrial permeability transition (MPT) cell death	**4**/204 (1.96%)	TP63 EYA2 CP LTF
Lysosomal cell death	**3**/233 (1.29%)	NR4A3 CTSV ATP6V0D2
Mitotic cell death	**21**/1701 (1.23%)	SERPINB3 TFF3 TP63 SOCS2 IL6 CD24 VCAM1 USP2 BTG2 EYA1 GADD45B FRZB BHLHE40 BIRC7 ADGB STOX1 DUSP1 WEE1 NNMT PIM2 FOXC1
Proliferation-associated cell death	**31**/1782 (1.74%)	CXCL13 GSTA2 POU2AF1 EGR1 CD69 CHL1 IL6 CD24 EGR2 TRIB1 NME5 VCAM1 SOCS3 IRF4 MAK CCL19 PFN1 DUSP5 CCN2 CTSV FRZB NFKBIZ SLC18A2 HBEGF ROPN1L FOSL1 BMPR1B CXCL2 NNMT HLA-DQA1 S100P

Transcriptomic analysis revealed that COPD upregulates genes associated with 12 distinct types of cell death, including: pyroptosis (21 genes), apoptosis (4 genes), necroptosis (3 genes), ferroptosis (8 genes), autosis (35 genes), autophagy (6 genes), parthanatos (4 genes), immunogenic cell death (62 genes), mitochondrial permeability transition (4 genes), lysosomal cell death (3 genes), mitotic cell death (21 genes), and proliferation-associated cell death (31 genes). Gene lists for each cell death category were obtained from the XDeathDB database (https://pcm2019.shinyapps.io/XDeathDB/).

Pathway enrichment analysis using Metascape (Supplementary Table 2) identified 43 significantly enriched pathways associated with the upregulated genes from the 12 regulated cell death categories. Notably, pyroptosis shared only a limited number of pathways with autosis, immunogenic cell death, mitotic cell death, and proliferation-associated cell death. In contrast, autophagy, parthanatos, ICD, and proliferation-associated cell death were largely characterized by distinct, non-overlapping pathway enrichments. Collectively, these findings demonstrate for the first time that COPD-induced lung injury involves not only the classical forms of cell death (apoptosis, necroptosis, pyroptosis, and ferroptosis), but also eight additional forms of regulated cell death, including autosis, autophagy-dependent cell death, parthanatos, immunogenic cell death, MPT-induced cell death, lysosomal-dependent cell death, mitotic cell death, and proliferation-associated cell death. This expanded our understanding of COPD pathogenesis and reveals novel mechanistic insights and suggests new potential therapeutic targets.

### 3.3 COPD promotes epithelial to mesynchymal transition and fibrosis

Lung fibrosis in COPD is increasingly recognized as a key pathological feature that contributes to small airway remodeling and progressive airflow limitation. In COPD, chronic inflammation, oxidative stress, and repeated epithelial injury promote fibroblast activation and extracellular matrix (ECM) deposition, leading to peribronchiolar fibrosis. This fibrotic remodeling narrows small airways, impairs lung elasticity, and often coexists with emphysematous destruction, together accelerating lung function decline (51, 52). Emerging evidence also suggests that specific molecular pathways such as EMT are central drivers of fibrosis in COPD (12). However, a comprehensive transcriptomic analysis specifically focused on defining the fibrotic landscape in COPD has not yet been conducted. To address this, we assessed transcriptomic changes in genes associated with EMT and fibrosis. As shown in Table 3, gene expression analysis revealed that COPD upregulates 36 of 1,153 EMT genes (3.12%) and 27 of 777 fibrosis-related genes (3.47%), while downregulated six EMT genes (0.52%), and ten fibrosis-related genes (1.29%). Metascape pathway analysis of these differentially expressed genes (Supplementary Table 3) identified significantly enriched pathways, with a dominant enrichment of proinflammatory signaling pathways.

**TABLE 3:** Chronic obstructive pulmonary disease (COPD) promotes epithelial-to-mesenchymal transition (EMT) and fibrotic remodeling in the lung.

Gene list	Upregulated/ downregulated	Number and percentage of differentially expressed genes	Differentially expressed genes
Epithelial–to-mesenchymal transition (EMT) genes	Upregulated genes	36/1,153 (3.12%)	KRT17 KRT5 CXCL6 TMPRSS4 TRIM29 TP63 CCN1 PROM1 TSPAN1 CD24 EGR2 B3GNT3 VCAM1 SOX2 CCR7 KRT15 CEACAM5 HAS2 LINC00312 GADD45B KLF10 CCN2 MUC20 IER3 SERPINE1 OSM FGFBP1 CXCL12 SLC22A4 HBEGF FOSL1 ELF3 NNMT AREG FOXC1 S100P
	Downregulated genes	6/1,153 (0.52%)	HMOX1 AOX1 S100A8 LIPG SRPX S100A9
Fibrosis-related genes	Upregulated genes	27/777 (3.47%)	HBB CXCL13 ALOX15 CXCL6 PTGS2 EGR1 PROM1 CD69 IL6 APLN CSF3 NR4A1 SOCS3 CCR7 CCL5 MUC5B HAS2 CCL19 DUSP5 LIF KSR1 SERPINE1 SLC22A4 HBEGF FOSL1 JUND AREG
	Downregulated genes	10/777 (1.29%)	DEFA3 DEFA1 S100A12 ABCC8 CD163 HMOX1 S100A8 TRIB3 S100A9 TTN

Transcriptomic analysis showed that COPD upregulated more EMT genes (36/1,155; 3.12%) compared to the downregulated EMT genes (6/1,155; 0.52%) and upregulated more fibrosis-related genes (27/778; 3.47%) than downregulated fibrosis-related genes (10/778; 1.29%). Gene sets were obtained from the following sources: COPD transcriptomic dataset: NIH-NCBI GEO database (GSE239897); EMT gene set: EMTome database (www.emtome.org); Fibrosis-associated genes: Previously published study of fibrosis in eight fibrotic diseases (PMID: 33519923).

COPD also modulated the gene expression of epithelial and mesenchymal genes where 259 of 5,769 epithelial cell genes (4.49%) and 26 of 901 mesenchymal cell genes (2.89%) were upregulated in COPD, COPD also downregulated 37 epithelial cell genes (0.64%) and ten mesenchymal cell genes (1.11%) (Supplementary Table 4). These findings indicate that COPD predominantly induces upregulations—rather than downregulation—of genes involved in epithelial and mesenchymal cell identity, while simultaneously promoting EMT and fibrosis. When integrated with data presented in Table 1, these results suggest that COPD promotes chronic inflammation, fibrogenesis, and tissue remodeling by reprogramming multiple cell types and activating inflammatory and profibrotic gene networks.

### 3.4 COPD induces lung tissue dysfunction through transcriptomic reprogramming and pathological trans-differentiation

**FIGURE 2:**
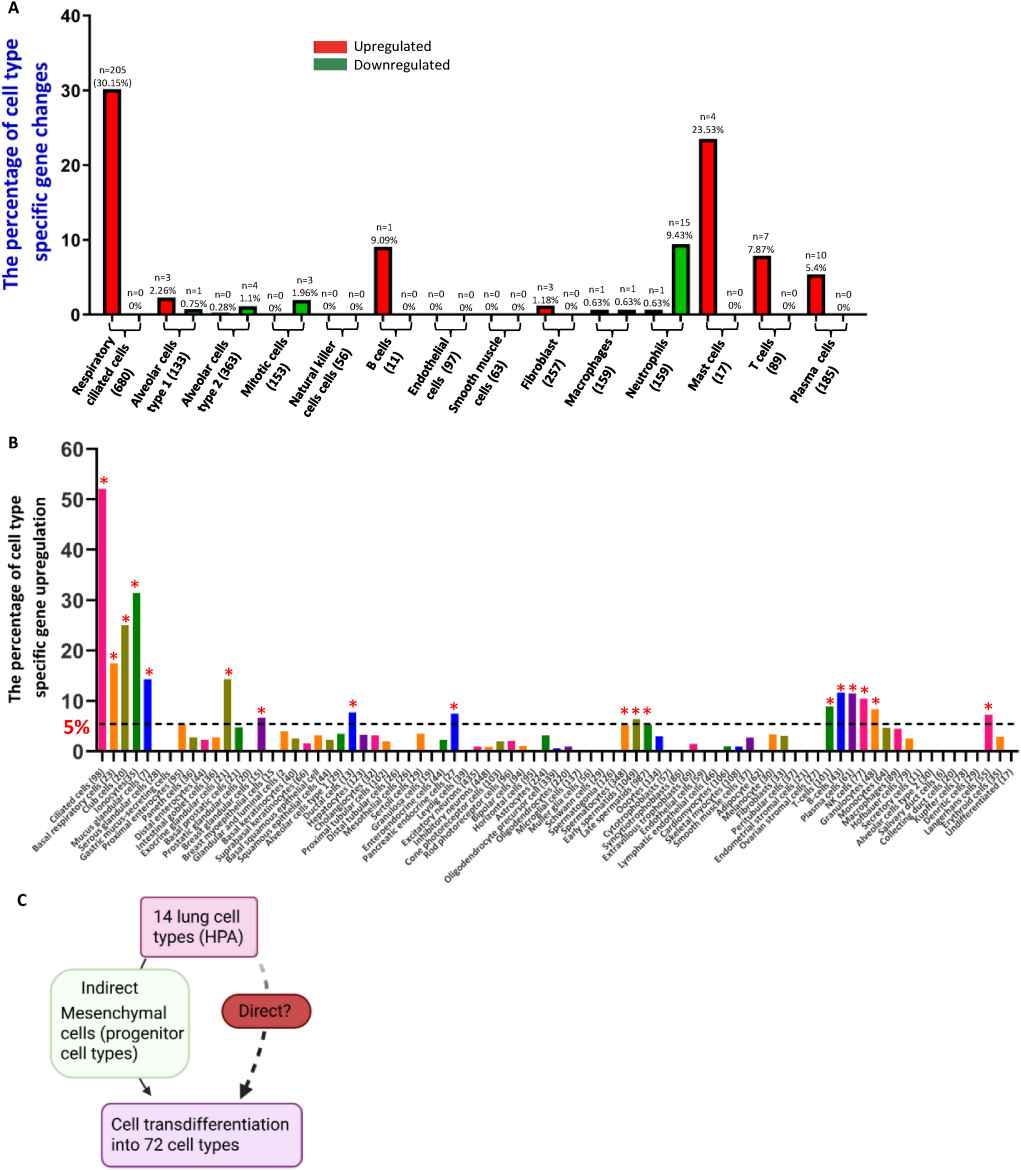
Chronic obstructive pulmonary disease (COPD) induces lung tissue damage, transcriptional reprogramming of resident cells, and pathological cell trans-differentiation. **(A)** Transcriptomic profiling demonstrated that COPD markedly alters the expression of cell-type–specific marker genes across 10 of 14 major human lung cell types. Increased marker expression was detected in respiratory ciliated cells, alveolar type I cells, B cells, mast cells, T cells, and plasma cells, whereas decreased expression was observed in alveolar type II cells, mitotic cells, and neutrophils. Cell-type–specific gene sets were obtained from the Human Protein Atlas (HPA). **(B)** COPD significantly increased the expression of cell-type–specific marker genes in 52 of 81 human cell types, with 18 cell types marked by * exhibiting > 5% marker gene enrichment. These included ciliated cells, basal respiratory cells, club cells, ionocytes, mucus glandular cells, proximal enterocytes, exocrine glandular cells, breast glandular cells, pancreatic endocrine cells, spermatocytes, early and late spermatids, T cells, B cells, plasma cells, NK cells, granulocytes, and Langerhans cells. **(C)** COPD promotes pathological cell trans-differentiation, likely through both direct injury-induced mechanisms and indirect inflammatory signaling, contributing to altered cellular identity and impaired tissue homeostasis.

We recently proposed that pathological trans-differentiation plays a critical role in the progression of chronic inflammation and cardiovascular diseases (24). In line with this concept, a recent single-cell RNA-Sequencing (scRNA-Seq) study by Sauler et al. (53) identified a subpopulation of alveolar epithelial type 2 (AT2) cells in COPD lungs with gene expression signatures indicative of metabolic dysregulation and impaired stress responses. The study also identified proinflammatory endothelial subtypes with increased CXCL chemokine signaling and macrophage subset enriched for metallothionein expression, particularly in advanced COPD. In support of these findings, other studies have documented the plasticity of lung epithelial cells in response to injury, such as the de-differentiation of secretory cells into basal cells and the capacity of Hopx^+^ alveolar type 1 (AT1) cells to generate AT2 following pneumonectomy (54). Based on this, we hypothesized that COPD alters the transcriptomes of multiple cell type-specific marker genes across diverse pulmonary cell populations. As illustrated in Figure 2A, the proportion of upregulated marker genes in COPD varied significantly across different lung cell types: 30.15% in respiratory ciliated cells, 23.53% in mast cells, 9.01% in B cells, 7.8% in T cells, 5.4% in plasma cells, 2.26% in AT1 cells, and 1.18% in fibroblasts. Using established methodologies (36, 55), we further examined cell type–specific gene expression across 81 human cell types curated by the Human Protein Atlas (HPA). As shown in Figure 2B, 52 of the 81 cell types demonstrated significant transcriptional upregulation in COPD lungs, with 18 showing more than a 5% increase in cell type–specific gene expression. These included ciliated cells, basal respiratory cells, club cells, ionocytes, mucus glandular cells, immune cell subsets (T cells, B cells, plasma cells, natural killer (NK) cells, granulocytes, and Langerhans cells), as well as non-respiratory cell types such as enterocytes, exocrine and breast glandular cells, pancreatic endocrine cells, and spermatogenic cells.

Together, these results suggest that COPD triggers extensive transcriptomic reprogramming in 10 out of 14 major lung cell types, likely through both direct injury and indirect signaling pathways, potentially involving mesenchymal cell-mediated mechanisms (Figure 2C). This reprogramming is characterized by the widespread upregulation of cell type-specific markers, offering mechanistic insights into how COPD drives tissue dysfunction and pathological cell trans-differentiation.

### 3.5 COPD enhances immune responses by downregulating inhibitory and stimulatory immune checkpoint ligands on alveolar macrophages

Immune checkpoints, typically expressed on T cells and CD4^+^Foxp3^+^ Treg, and their corresponding ligands expressed on APCs, are critical membrane-bound regulators of immune homeostasis. These checkpoint pathways mediate essential cell-cell interactions between T cells (including Tregs) and APCs (32, 37, 56, 57). Immune checkpoint inhibitors have become standard-of-care therapy for various malignancies, particularly lung cancer, the most common comorbidity in patients with COPD (21). Additionally, emerging immunotherapies such as adoptive T cell strategies, including Treg-based treatments, are being explored for chronic respiratory diseases like severe asthma and COPD (58). Given this context, we hypothesized that COPD alters the expression of immune checkpoint ligands, particularly on alveolar macrophages. As shown in Table 4, COPD was associated with significant downregulation of 19 out of 51 known immune checkpoint ligands [curated in our recent review (37)] in alveolar macrophages of smokers with COPD (28, 30, 31) relative to healthy non-smokers. This included 11 inhibitory, seven stimulatory, and one dual-function ligand. When compared to healthy smokers, seven ligands were significantly downregulated in COPD (four inhibitory, two stimulatory, and one dual-function ligand). In contrast, the difference between healthy smokers and healthy non-smokers involving the downregulation of four inhibitory ligands, two stimulatory ligands, and one dual function ligand.

**TABLE 4:** Chronic obstructive pulmonary disease (COPD) may enhance immune activation by downregulating immune checkpoint ligand expression in alveolar macrophages.

Comparison group (GSE13896)	Upregulated immune checkpoint ligands	The number of inhibitory/ stimulatory genes	Downregulated immune checkpoint ligands	The number of inhibitory/ stimulatory genes
Healthy smokers compared to healthy non-smokers	TNFSF8 (I) TNFSF14 (S)	2 (1 Inhibitory, 1 stimulatory)	CD80 (RD) CD2 (S) CD96 (I) VTCN1 (I) SLAMF1 (S) CTLA4 (I) TNFRSF25 (I)	7 (4 Inhibitory, 2 stimulatory, 1 dual function)
Smokers with COPD compared to healthy non-smokers	TNFSF8 (I) SIGLEC15 (I)	2 Inhibitory	PVR (RD) TNFRSF4 (S) CD2 (S) CDH1 (I) SELPLG (I) NECTIN3 (I) CD96 (I) SIRPA (I) ICOS (S) CD70 (S) SLAMF1 (S) VTCN1 (I) TNFRSF25 (I) CTLA4 (I) ICOSLG (S) CD274 (I) TNFRSF18 (S) HLA-G (I) PDCD1LG2 (I)	19 (11 Inhibitory, 7 stimulatory, 1 dual function)
Smokers with COPD compared to healthy smokers	CD80 (S/I) CD58 (S)	2 (1 Dual function, 1 stimulatory)	CD276 (S) PVR (RD) SIRPA (I) CDH1 (I) SELPLG (I) NECTIN3 (I) TNFRSF4 (S)	7 (4 Inhibitory, 2 stimulatory, and 1 dual function)

Transcriptomic analysis of alveolar macrophages from COPD patients revealed significant downregulation of 11 inhibitory (i), 7 stimulatory (s), and one dual-function immune checkpoint ligands out of a curated panel of 51 immune checkpoint ligands previously reported in our recent review (PMID: 39926714). Comparative analysis showed that smokers with COPD exhibited reduced expression of 19 immune checkpoint ligand genes relative to normal non-smokers, and 7 immune checkpoint ligand genes relative to normal smokers. These findings suggest a potential mechanism of increased immune activation and dysregulation in the COPD lung microenvironment. Data were obtained from the NIH-NCBI GEO database (accession: GSE13896), and differential gene expression was determined using a cutoff of log2 fold change > 1 and *P* < 0.05.

These findings indicate that COPD results in a broader and more pronounced downregulation of inhibitory immune checkpoint ligand expression than stimulatory ones. This preferential loss of inhibitory signaling on alveolar macrophages may effectively “release the brakes” on immune activation, promoting exaggerated inflammatory responses in the lung and contributing to disease progression.

### 3.6 PVR (CD155) downregulation in lung tissue and alveolar macrophages in severe emphysema and COPD

**FIGURE 3:**
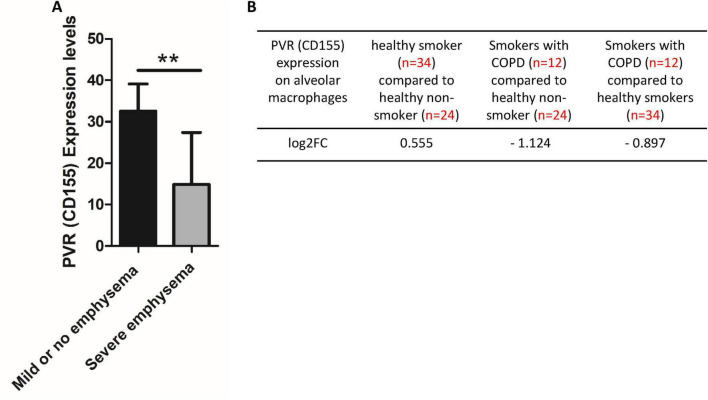
Chronic obstructive pulmonary disease (COPD) downregulates the expression of the inhibitory immune checkpoint ligand poliovirus receptor (PVR/CD155) in emphysematous lung tissue and alveolar macrophages of COPD. **(A)** PVR expression was significantly reduced in lung tissue from smokers with severe emphysema (*n* = 18) compared to those with mild or no emphysema (*n* = 12), suggesting a potential role of PVR downregulation in the pathogenesis of COPD-related emphysema. Data were obtained from the NIH-NCBI GEO database (GSE1650). **(B)** PVR (CD155) expression was also significantly downregulated in alveolar macrophages from smokers with COPD relative to both normal non-smokers and normal smokers. No significant difference in PVR expression was observed between normal non-smokers and normal smokers, indicating that the reduction in PVR is specifically associated with COPD rather than smoking alone. **P < 0.01.

Building on our recent comprehensive analysis of immune checkpoint receptor-ligand pairs in aortic diseases, which identified CD155 (poliovirus receptor, PVR) as key immunosuppressive ligands (38), and supported by emerging single-cell RNA sequencing studies highlighting a critical role for PVR in mediating immunosuppression within the tumor microenvironment (59), we hypothesized that PVR expression is similarly downregulated in progressive emphysema. To investigate this, we analyzed transcriptomic data from lung tissues of emphysema patients using the publicly available dataset GDS737. As shown in Figure 3A, PVR expression was significantly reduced in lung tissues from smokers with severe emphysema (*n* = 18) compared to those with mild or no disease (*n* = 12), suggesting that PVR loss may contribute to disease severity. Furthermore, analysis of alveolar macrophage transcriptomic profiles (GSE13896; log_2_FC > 1 and *P* < 0.05) revealed consistent patterns. PVR expression was significantly downregulated in alveolar macrophages from smokers with COPD (*n* = 12), showing a -1.124 log_2_FC compared to healthy non-smokers (*n* = 24) and -0.897 log2FC relative to healthy smokers (*n* = 34) (Figure 3B). Notably, PVR expression in alveolar macrophages from healthy smokers was not significantly altered relative to healthy non-smokers (0.555 log_2_FC). These data suggest that PVR downregulation in both lung tissue and alveolar macrophages is a COPD-specific phenomenon, potentially contributing to its inflammatory pathogenesis. Further mechanistic studies are warranted to explore the therapeutic relevance of targeting PVR.

### 3.7 PVR overexpression suppresses proinflammatory gene expression in alveolar and interstitial macrophages in COPD

In addition to its immunosuppressive role through intercellular (outward) interaction with the immunosuppressive immune checkpoint receptor TIGIT (T cell immunoreceptor with Ig and ITIM domains) expressed on the plasma membranes of T cells and Treg, we hypothesized that PVR may also exert intracellular (inward) immunosuppressive effects within APCs such as macrophages. To explore this, we analyzed a multiple public transcriptomic datasets, including those from COPD lung tissues (GSE239897) (4), CD155-overexpression (CD4 T^+^ cells stimulated with CD155-expressing L cells) (GSE194293), alveolar macrophages from COPD patients (GSE13896) (60), and a smoking/COPD emphysema lung samples (GDS737) (61).

As summarized in Table 5, PVR overexpression significantly downregulated 13 genes that are upregulated in COPD lungs, 16 COPD-associated genes in sputum, 13 genes upregulated in healthy smokers, and 19 or 17 genes in COPD smokers (relative to healthy non-smokers or healthy smokers, respectively). These gene changes (log_2_FC > 1 and *P* < 0.05) suggest a broad anti-inflammatory effect of PVR.

**TABLE 5:** PVR (CD155) overexpression suppresses gene programs upregulated in chronic obstructive pulmonary disease (COPD).

Comparison groups	PVR overexpression inhibited COPD upregulated genes	Metascape pathway analysis showed the top pathways of the overlapped genes
COPD compared to non-COPD lung tissues (GSE239897) Log2FC > 1, *n* = 11	(**13**) CCDC17 SPEF2 DUSP5 ODAD4 NR4A2 CCN1 RGS1 AK7 ANKRD36BP2 UBD SOCS2 ATF3 APLN	VEGFA-VEGFR2 signaling Positive regulation of apoptotic process Heart development Brain development
COPD (n-72) compared to healthy controls sputum (*n* = 15), Log2FC > 1	(**16**) TNFAIP3 FKBP1AP1 MAFF SEMA6A SEZ6 ABCG1 ZBTB20 AK4 CES3 MYO1H CAMK2B ANKRD18DP XAF1 DUSP8 RNF165 ADM	Positive regulation of lipid biosynthetic process Myometrial relaxation and contraction pathways Nuclear receptors meta pathway Regulation of angiogenesis
Healthy smokers compared to healthy non-smokers	(**13**) N4BP2L2-IT2 DOCK3 PGAP1 ENO2 TMEM163 ZNF395 COL1A1 PNPLA3 KCTD15 GBP6 HES4 TNS1 CD22	–
Smokers with COPD compared to healthy non-smokers	(**19**) ZC3HAV1L GPR146 MYC TEX14 PCSK6 DDX60L TMEM163 PTCH1 RGS1 EPS8 CRABP2 PNPLA3 SLCO4C1 KCTD15 ETV5 DNAJC12 TNS1 CD22 ALDH1L2	Negative regulation of cell division Male gamete generation Monocarboxylic acid metabolic process
Smokers with COPD compared to healthy smokers	(**17**) ZC3HAV1L RAB6B ZBTB20 PCSK6 GAB1 CNKSR3 RGS1 LOC100506274 CD80 TREML2 SLCO4C1 APBB2 ETV5 NIM1K RGMA SLC8A3 ASS1	Regulation of protein phosphorylation Transport of small molecules Neuron projection development Response to bacterium

Overexpression of PVR (CD155) significantly downregulated genes that are otherwise upregulated in lung tissue, sputum, and alveolar macrophages of COPD patients. Data were obtained from the following GEO datasets: COPD lung tissue (GSE239897,), CD4^+^ T cells stimulated with CD155-expressing L cells (GSE194293), alveolar macrophages from COPD patients (GSE13896), and COPD emphysema/smoking dataset (GDS737). Differential expression was considered significant at log*2* fold change > 1 and *p* < 0.05.

Further analysis (Table 6) showed that PVR overexpression suppressed 49 proinflammatory genes expressed in CD14^+^IL-6R*^high^* monocytes from lungs of COPD patients (GSE265853), which are key contributors to COPD exacerbation (60). Metascape pathway enrichment revealed these genes are involved in several biological pathways.

**TABLE 6:** PVR overexpression–mediated suppression of pro-inflammatory gene programs in CD14^+^IL-6Rhigh monocytes in COPD.

PVR overexpression inhibited 49 CD14^+^IL6R^high^ monocyte upregulated genes	Metascape pathway analysis showed the top pathways of the 49 overlapped genes
MORN3 CAPN3 PSD3 MATCAP1 RILP ARHGAP39 RGMB RAB13 RCN3 ABCG1 SPINT1 FMNL2 CDK18 CA2 SHB TANC2 ZNF442 TEX14 MAMDC4 LINC02863 CABP4 CFB LRP1 KLHL29 PTK2 TCF7L2 LOC653513 NOXA1 MGAT3 SCD SAMD4A TMEM132A PKD2L1 PNPLA3 SAMD9L ZBED3 CACNA1F CD274 TMEM74B PTPRO AKAP12 IL1A LOC105372814 ALDH2 AXL OASL IGFBP2 DTNA SERPINA1	Affected pathways in Duchenne muscular dystrophy Regulation of Wnt signaling pathway Positive regulation of cell activation Response to peptide hormone Detection of external stimulus Positive regulation of protein secretion Negative regulation of protein metabolic process VEGFA-VEGFR2 Pathway Regulation of Insulin-like Growth Factor (IGF) transport and uptake by Insulin-like Growth Factor Binding Proteins (IGFBPs)

PVR overexpression suppressed 49 pro-inflammatory genes expressed in CD14^+^IL-6Rhigh monocytes—identified as key contributors to COPD exacerbation. These genes were enriched in 10 signaling pathways. Data was retrieved from GEO dataset GSE265853.

In addition, PVR overexpression significantly downregulated 76 genes associated with M1 (proinflammatory) macrophage polarization (M1 versus (vs.) M0), 14 genes associated with M2 (anti-inflammatory) macrophage polarization (M2 vs. M0), and 71 genes expressed more highly in M1 compared to M2 macrophages (M1 vs. M2). This yields an M1/M2 gene suppression ratio: 76/14 = 5.43, suggesting preferential inhibition of proinflammatory M1-associated gene expression. Pathway analysis revealed that 10 of the top 18 signaling pathways affected by PVR overexpression were associated with M1 macrophage genes. These included interferon-α/β signaling, type II interferon signaling, interleukin signaling, etc., which contrast with M2-associated pathways such as vitamin D receptor pathway and the negative regulation of cell-cell adhesion (Table 7).

**TABLE 7:** PVR overexpression modulates M1 and M2 macrophage polarization gene programs.

Comparison GSE5099, Log2FC > 1	PVR overexpression downregulated genes (938), Log2FC > 1	Top pathways
M1 vs. M0 upregulated genes (76/812)	TNFAIP3 LAMB3 CHI3L2 HMGCS1 NDRG1 SPHK1 SOCS1 OAS2 LOXL2 PARP12 RNF24 PSD3 PHF11 TBX21 TTC28 BSPRY DUSP5 IL10RA TRIM22 RIPK2 CDK18 PAM PMEPA1 NAMPT ZBTB10 CFB IGF2BP2 OAS3 IFIT5 MUC1 IFIH1 ZBTB32 CKB COL1A1 DHX58 CLIC4 CD80 INSIG2 LAMP3 JUN IRF8 TMCC2 FOSL2 RGS16 GRB10 PHLDA2 MX2 ISG15 G0S2 HERC6 HERC5 ISG20 XAF1 BCL2L14 USP18 IRF7 IL1RN OAS1 DDX60 KLF4 CCL20 IFI44 SOCS2 OASL MX1 ATF3 PNMA2 IFIT2 IFIT1 IFI44L RSAD2 IFIT3 FERMT2 SERPINA1 ASS1 ADM	Interferon alpha/beta signaling response to type I interferon response to bacterium Antiviral mechanism by IFN-stimulated genes Modulation of host responses by IFN-stimulated genes Immune response to tuberculosis Type II interferon signaling Network map of SARS CoV 2 signaling Signaling by Interleukins Signaling by Interleukins
M2 vs. M0 upregulated genes (14/179)	TNFAIP3 SOCS1 RNF24 PSD DUSP5 MYC JAG1 CKB G0S2 PDGFB IL1RN KLF4 DACT1 SERPINA1	Vitamin D receptor pathway negative regulation of cell-cell adhesion positive regulation of miRNA transcription negative regulation of intracellular signal transduction negative regulation of cell activation Signaling by Interleukins inflammatory response positive regulation of apoptotic process
M1 vs. M2 upregulated genes (71/746)	TNFAIP3 LAMB3 CHI3L2 HMGCS1 SPHK1 SOCS1 OAS2 LOXL2 PLAUR PARP12 PHF11 TBX21 TTC28 BSPRY DUSP5 IL10RA TRIM22 RIPK2 CDK18 PAM PMEPA1 NAMPT CFB IGF2BP2 OAS3 IFIT5 MUC1 IFIH1 ZBTB32 CKB RGS1 COL1A1 DHX58 CLIC4 CD80 LAMP3 JUN IRF8 TMCC2 FOSL2 RGS16 GRB10 PHLDA2 MX2 ISG15 G0S2 HERC6 HERC5 ISG20 XAF1 BCL2L14 USP18 IRF7 IL1RN OAS1 DDX60 CCL20 IFI44 SOCS2 OASL MX1 ATF3 PNMA2 IFIT2 IFIT1 IFI44L RSAD2 IFIT3 FERMT2 ASS1 ADM	Interferon alpha/beta signaling response to type I interferon response to bacterium Antiviral mechanism by IFN-stimulated genes Modulation of host responses by IFN-stimulated genes Immune response to tuberculosis Type II interferon signaling Network map of SARS CoV 2 signaling Signaling by Interleukins positive regulation of apoptotic process

PVR overexpression downregulated 76 genes associated with M1 macrophage polarization (M1 vs. M0), 14 genes related to M2 polarization (M2 vs. M0), and 71 genes with higher expression in M1 relative to M2 macrophages (M1 vs. M2). Signaling pathways associated with M1 macrophage-related genes differed markedly from those linked to M2 macrophages. Data was sourced from macrophage polarization dataset GSE5099.

As shown in Table 8, COPD was associated with the upregulation of seven genes enriched in interstitial macrophages (IMs) relative to alveolar macrophages (AMs) (62), three genes elevated in small IMs compared to small AMs, and three genes specific to large AMs. Conversely, among genes classified as “COPD downregulated,” AMs exhibited higher expressions of *IL-1R2* and *IL-18R1* compared to IMs, while small AMs expressed higher levels of *IL-1R2*, *S100A8*, and *S100A9* compared to large AMs. Moreover, COPD-AMs showed increased expression of ten genes (so-called “downregulation in IMs”), including *NLRP3 (*NOD-like receptor protein 3*)* (48), *CCL20* *(*C-C motif chemokine ligand 20*)*, *INHBA* (inhibin subunit beta A), *ICAM1 (*intercellular adhesion molecule 1*)*, *IL-1*β (interleukin-1β) (63), *IL18R1*, *IL1A*, *CXCL1* (C-X-C Motif Chemokine Ligand 1) (64), *SERPINE1* (Serpin family E member 1), and *TJP1* (Tight Junction Protein 1) in AMs compared to IMs, upregulation of four genes (so-called “downregulation in small IMs”), including *CCL20*, *ICAM1*, *IGF1* (insulin like growth factor 1), and *CXCL1* in small AMs compared to small IMs, and upregulation of seven genes (so-called “downregulation in large IMs”), including *NLRP3*, *CCL5*, *CXCL5*, *INHBA, ICAM1, IL1A*, and *HGF* (hepatocyte growth factor) in small AMs compared to large AMs. PVR overexpression was found to suppress the expression of eight genes in IMs including *IL-1RL1 (*interleukin 1 receptor like 1*), MUC1* (Mucin 1, cell surface associated), *CLDN1* (Claudin 1), *IL-1A, VEGFA* (vascular endothelial growth factor A), *OAS1* (2’-5’-Oligoadenylate Synthetase 1), *CCL20*, and *IL-13*; three genes in small IMs such as *IL-1L1, VEGFA*, and *CCL20*; eight genes in large AMs, including *IL-1RL1, MUC1, IFIH1* (interferon induced with helicase C domain 1), I*L-1A, VEGFA, IRF7* (interferon regulatory factor 7), *MX1* (MX dynamin like GTPase 1), and *SERPINA1*. Together, these findings demonstrate that PVR exerts potent anti-inflammatory effects across alveolar and interstitial macrophage subsets in COPD, supporting its role as a central immunoregulatory checkpoint in lung inflammation. These findings highlight PVR as a promising therapeutic target for modulating macrophage-driven inflammation in chronic lung diseases.

**TABLE 8:** COPD upregulated genes enriched in interstitial macrophages (IMs) and alveolar macrophage (AM) subsets, including: 4 IM-over-AM genes, 5 small IM-over-small AM genes, 2 large AM-specific genes, and 1 small AM-specific gene, while downregulating 1 small AM gene.

Four COPD macrophage subsets including small interstitial macrophages, large interstitial macrophages, small alveolar macrophages and large alveolar macrophages (PMID: 28769058)	COPD (GSE239897)		COPD alveolar macrophages		PVR overexpression	
	Upregulated	Downregulated	Upregulated	Downregulated	Upregulated	Downregulated
Inflammatory-related genes that are significantly expressed in interstitial macrophages compared to alveolar macrophages from COPD patients (67 genes).	IL6 CSF3 CCL11 MUC5B CCL3 LTF SERPINE1	IL1R2 IL18R1	TGFB2 IFNA1 OCLN	NLRP3 CCL20 INHBA ICAM1 IL1B IL18R1 IL1A CXCL1 SERPINE1 TJP1	NQO1	IL1RL1 MUC1 CLDN1 IL1A VEGFA OAS1 CCL20 IL13
Inflammatory related genes that are significantly expressed in small interstitial macrophages compared to small alveolar macrophages from COPD patients (17 genes).	MUC5B CCL3 LTF	–	TGFB2	CCL20 ICAM1 IGF1 CXCL1	NQO1	IL1RL1 VEGFA CCL20
Inflammatory related genes that are significantly expressed in large alveolar macrophages compared to small alveolar macrophages from COPD patients (42 genes).	CCL22 FOS CCL5	IL1R2 S100A8 S100A9	–	NLRP3 CCL5 CXCL5 INHBA ICAM1 IL1A HGF	TLR5	IL1RL1 MUC1 IFIH1 IL1A VEGFA IRF7 MX1 SERPINA1

COPD-associated AMs also showed increased expression of NLRP3, VEGFA, and ICAM1, and 2 additional genes enriched in small IMs. PVR overexpression suppressed 2 IM genes, 3 small IM genes, and 1 large AM gene. Regulatory T cells (CD4^+^Foxp3^+^ Tregs) were shown to inhibit expression of 10 IM genes, 10 small IM genes, 2 large AM genes, and 8 small AM genes.

### 3.8 COPD weakens immunosuppressive Tregs by altering the transcriptional profiles of distinct Treg gene subsets

According to the World Health Organization, cigarette smoking is the leading cause of COPD.^4^ Previous studies have demonstrated a significant reduction in CD4^+^CD25^+^FoxP3^+^ Tregs in smokers compared to non-smoker healthy controls, suggesting impaired Treg-mediated immunosuppression in COPD. Through RNA-sequencing analysis, we have previously shown that both cigarette smoke and its combination with morphine profoundly alter Treg transcriptomes (28, 30, 31). Based on these findings, we hypothesized that COPD modifies the transcriptomic landscape of CD4^+^Foxp3^+^ Tregs, contributing to functional impairment. Our transcriptomic analysis (Table 9 and Supplementary Figures 1A–E) revealed that COPD upregulates 19 and downregulates six human Treg genes from the HPA database.

**TABLE 9:** COPD alters the transcriptional landscape of distinct regulatory T cell (Treg) subsets.

Regulatory T cells (Tregs) related datasets		Human lungs of COPD patients (GSE239897)	
		Upregulated genes (959)	Downregulated genes (414)
Treg genes (431, HPA)		19	6
Smoke Tregs compared to non-smoke Tregs (GSE198210)	Upregulated (515)	8	2
	Downregulated (542)	55	18
FoxP3 + compared to FoxP3- Tregs GSE164460	Upregulated (734)	19	5
	Downregulated (403)	6	3
Resolving lung Tregs after LPS treatment vs. control lung Tregs, GSE104088	Upregulated (109)	0	4
	Downregulated (103)	3	0
TIGIT + compared to TIGIT-(GSE56299)	Upregulated (599)	10	6
	Downregulated (174)	4	0

COPD induced significant transcriptional changes in genes associated with general Treg identity, including upregulation of 19 genes and downregulation of 6 genes. The general Treg gene list was curated from the Human Protein Atlas (HPA). Among genes associated with smoking-induced Tregs (“smoke Tregs”), COPD resulted in the downregulation of 55 genes. Within the FoxP3^+^ Treg-specific gene subset, 19 genes were upregulated and 5 were downregulated in COPD. Additionally, COPD upregulated 6 and downregulated 3 genes that are normally suppressed in FoxP3^+^ Tregs. In the lung Treg subset previously shown to be modulated by lipopolysaccharide (LPS), COPD upregulated 3 genes typically downregulated by LPS and downregulated 4 genes typically upregulated by LPS. Within the TIGIT^+^ Treg-associated gene set, COPD upregulated 10 genes normally elevated in TIGIT^+^ Tregs and 4 genes typically downregulated, while downregulating 6 genes that are normally upregulated in this subset.

Furthermore, our analysis of previously published mouse Treg RNA-Seq dataset from a 2-month cigarette smoke exposure model (28, 30, 31) revealed that COPD upregulated eight genes previously identified as “smoke-upregulated” Treg genes and 55 of Treg inhibited genes “smoke-downregulated” Treg genes. These 55 genes are associated with Treg weakening, consistent with the hypothesis that COPD promotes Treg dysfunction and persistent inflammation. COPD also upregulated 19 and downregulated five Foxp3^+^ Treg-specific genes, including *BIRC5, OSGIN1, IL-1R2, SPOCK2*, and *TTN* (Titin). Furthermore, COPD downregulated four lipopolysaccharide (LPS)-responsive genes in lung Tregs—*HMOX1, CLEC4E, IL-1R2*, and *F2RL3—*which are involved in antimicrobial immunity. In addition, six TIGIT + Treg genes—*CCDC41, FKBP5, HMOX1, IL-1R2, SPOCK2*, and *IL-18R1* were downregulated in COPD, indicating compromised immunosuppressive function.

The biological significances of these downregulated Treg genes have been previously described: *BIRC5* (survivin) is upregulated in superTregs, which are more immunosuppressive than defective Treg and promote superTreg proliferation (65). *OSGIN1* (Oxidative stress induced growth inhibitor 1) is a signature marker of tissue-resident memory T cells (Trm) (66). *IL-1R2* acts as a decoy receptor that attenuates Treg activation (67). *SPOCK2* (SPARC (Osteonectin), Cwcv and Kazal like domains proteoglycan 2) interact with Foxp3 to facilitate Treg communication (68). *TTN*, regulate T lymphocyte trafficking (69). *HMOX1* (Heme oxygenase 1), induced by Foxp3, is essential for Foxp3^+^ Treg-mediated immune suppression (70). *CLEC4s* (C-type lectin domain family 4 member E) expression is positively correlated with immune cell infiltration (B cells, CD8^+^ T cells, CD4^+^ T cells, macrophages, neutrophils, and dendritic cells) (71). *F2RL3* (F2R like thrombin or trypsin receptor 3) modulates CD25, CD62L, and CD73 expression, Treg stability, and suppressive function through phosphorylation of FoxO1 and negative regulation of PTEN and FoxP3 (72). *CCDC41* (Centrosomal Protein 83) also known as Cep83 is involved in membrane contact site formation during ciliogenesis and is required for cytotoxic T lymphocyte secretion, potentially facilitating Treg migration and secretion (73). *FKBP5* (FKBP prolyl isomerase 5) has been linked to Treg dysfunction; high levels of FKBP51s^+^ Tregs were observed in non-responders to prototypic immunosuppressive immune checkpoint cytotoxic T-lymphocyte associated protein 4 (CTLA-4) blockade (ipilimumab), suggesting impaired CTLA-4 signaling in these cells (74), suggesting that FKBP5 may decrease the expression of CTLA4 and present a CTLA4 signaling weakening. *IL-18R1 (*Interleukin 18 receptor 1) is critical for Foxp3^+^ Treg cell-mediated control of inflammation, where it promotes the expression of key Treg effector molecules (75). After removing redundant entries, we identified 11 unique Treg-specific genes (*BIRC5, OSGIN1, IL-1R2, SPOCK2, TTN, HMOX1, CLEC4E, F2RL3, CCDC41, FKBP5*, and *IL-18R1*) consistently downregulated in COPD. These results support the hypothesis that COPD-induced transcriptional reprogramming of Tregs contributes to their dysfunction, thereby facilitating chronic pulmonary inflammation.

### 3.9 COPD weakens the functions of Treg, and other cell-secreted immunosuppressive cytokine IL-10 by downregulating IL-10-induced immunosuppressive genes

It has been reported that COPD progression is associated with elevated serum levels of the proinflammatory cytokine IL-17 and reduced levels of anti-inflammatory cytokines IL-10 and IL-35 (76). IL-10, like IL-35, plays a crucial role in immune tolerance and supports Treg function in the COPD lung environment (77). We hypothesized that COPD impairs the function of IL-10—secreted by Treg and other immune cell—by modulating IL-10-induced transcriptional responses. To test this hypothesis, we analyzed five publicly available datasets from the NCBI-GEO database. As shown in Table 10 and Supplementary Figures 2A–E, in the CD4^+^ T cell stimulated with IL-10 (GSE17199) dataset, COPD upregulated 16 IL-10-induced genes in CD4^+^ T cells, suggesting compensatory activation of IL-10 pathways during inflammation. Nevertheless, COPD also upregulated nine IL-10–suppressed genes and downregulated three IL-10-induced genes such as *TRIB3* (Tribbles pseudokinase 3), *DDIT4* (DNA damage inducible transcript 4), and *PSAT1* (phosphoserine aminotransferase 1). In a second IL-10-treated CD4^+^ T cell dataset (GSE198963), COPD similarly downregulated *RIPOR3* (RIPOR family member 3), another IL-10–upregulated gene and reversed the repression of five genes suppressed by IL-10. We then explored IL-10 regulation in innate immune cells in COPD, we analyzed a transcriptomic dataset of IL-10-treated monocytes (GSE43700) and found that IL-10 downregulated 29 COPD-upregulated genes and upregulated 11 COPD upregulated genes, including *GLT1D1 (*glycosyltransferase 1 domain containing 1)*, TRIB3, HK3 (*Hexokinase 3)*, SIGLEC16* (sialic acid binding Ig like lectin 16), *HMOX1, P2RY1* (purinergic receptor P2Y1), *SLCO4A1 (*solute carrier organic anion transporter family member 4A1)*, CD163, DDIT4, PSAT1*, and *SIGLEC10 (*sialic acid binding Ig like lectin 10). These genes are enriched in metabolic and homeostatic pathways, highlighting IL-10’s role in immunometabolic regulation—disrupted in COPD.

**TABLE 10:** IL-10 Counteracts COPD-associated proinflammatory gene expression across multiple immune cell types.

IL-10 related datasets		Human lungs of COPD patients (GSE239897)	
		Upregulated genes (959)	Downregulated genes (414)
CD4 + T cell stimulated with IL-10 (GSE17199)	Upregulated (436)	16	3
	Downregulated (355)	9	1
CD4 + T cell stimulated with IL-10 (GSE198963)	Upregulated (191)	13	1
	Downregulated (77)	5	0
Peripheral blood mononuclear cells derived monocytes treated with IL10 (GSE43700)	Upregulated (1,313)	31	11
	Downregulated (1,641)	29	7
Human monocyte derived macrophages stimulated with LPS and IL-10 blocking antibody (GSE181250)	Upregulated (346)	23	3
	Downregulated (330)	6	3
Monocyte derived DC cell stimulated with IL-10 (GSE180761)	Upregulated (1,872)	38	13
	Downregulated (1,222)	24	6

IL-10 modulates the expression of genes upregulated in COPD lung tissue by altering transcriptional responses in CD4^+^ T cells, monocytes/macrophages, and dendritic cells (DCs). Gene expression data were analyzed from publicly available transcriptomic datasets (NCBI GEO), with differential expression defined as log*2* fold change > 1 and *P* < 0.05. L-10 treatment of CD4^+^ T cells (GSE17199) downregulated 9 genes upregulated in COPD lung tissue (GSE239897). In an independent dataset (GSE198963), IL-10 treatment of CD4^+^ T cells downregulated 5 additional COPD-upregulated lung genes. IL-10 treatment of peripheral blood mononuclear cell (PBMC)-derived monocytes (GSE43700) suppressed the expression of 29 COPD-upregulated genes. Conversely, IL-10 blockade in human monocyte-derived macrophages (GSE181250) led to increased expression of 23 genes upregulated in COPD lung tissue. IL-10 treatment of monocyte-derived DCs (GSE180761) downregulated 24 COPD-upregulated lung genes. These results highlight IL-10 as a key immunoregulatory cytokine capable of reversing COPD-associated transcriptional changes across diverse immune cell subsets.

To further elucidate IL-10-regulated transcriptional programs in macrophages relevant to COPD, we analyzed a transcriptomic dataset, in which human monocyte-derived macrophages were primed with macrophage colony-stimulation factor (M-CSF), stimulated with LPS, and subsequently treated with an IL-10 blocking antibody (GSE181250). IL-10 inhibition upregulated 23 COPD-upregulated genes, suggesting these genes may be normally suppressed by IL-10 under homeostatic conditions. COPD also downregulated three IL-10 blocking downregulated (IL-10 promoted) genes such as *FKBP5* (FK506-binding protein prolyl isomerase 5)*, SLCO4A1*, and *HMOX1*, indicating that IL-10 may promote their expressions in healthy macrophage responses. We also analyzed a transcriptomic dataset of monocyte-derived DC stimulated with IL-10 (GSE180761). IL-10 suppressed 24 COPD-upregulated genes, while COPD downregulated 13 IL-10-upregulated genes, including *GLT1D1* (glycosyltransferase 1 domain containing 1), *BIRC5, HK3, HMOX1, OSGIN1, LOC105377771, IL-18R1, KIF2C* (kinesin family member 2C), *SLCO4A1*, *CD163, SPOCK2, SIGLEC11*, and *S100A9* (S100 calcium binding protein A9).

Pathway enrichment analysis of these overlapping genes revealed significant association with RHO GTPase effector signaling, Leishmania infection, and inflammatory response pathways, underscoring the immunoregulatory role of IL-10 in counteracting inflammatory gene expression programs that are dysregulated in COPD (Supplementary Figures 2A–E).

Taken together, our data have demonstrated that: *first*, anti-inflammatory cytokine IL-10 modulates the transcriptomes of monocytes, macrophages and DCs in COPD more than that in CD4^+^ T cells; *second*, some IL-10 upregulated genes are induced in COPD, suggesting that IL-10 pathways are responsive to COPD inflammation induction; *third*, COPD downregulates some IL-10 pathways genes, suggesting that COPD weakens IL-10 signaling for disease progression; and *fourth*, IL-10 inhibits some COPD upregulated genes, suggesting that IL-10 fulfills its anti-inflammatory and immunosuppressive functions in COPD.

### 3.10 PVR overexpression shares immunosuppressive gene signature with IL-10 in CD4^+^ T cells and myeloid cells

We hypothesized that the immunosuppressive function of PVR (CD155) may partially overlap with IL-10-mediated transcriptional programs in both CD4^+^ T cells and myeloid cells. To assess this hypothesis, we conducted a comparative transcriptomic analysis using publicly available datasets obtained from the NCBI-GEO database. In CD4^+^ T cells (Table 11 and Supplementary Figures 3A–E), no genes were commonly upregulated by both PVR overexpression and IL-10 stimulation, indicating distinct activation programs. However, six genes upregulated by PVR were downregulated by IL-10, and 21 genes downregulated by PVR were upregulated by IL-10. These findings highlight divergent regulatory effects exerted by PVR and IL-10 in CD4^+^ T cells. Despite this divergence, nine genes—*IL-1A, YPEL5* (Yippee like 5), *TRANK1* (tetratricopeptide repeat and Ankyrin repeat containing 1), *ZBP1* (Z-DNA Binding Protein 1), *MX2* (MX Dynamin Like GTPase 2), *IFI6* (interferon alpha inducible protein 6), *HERC5* (HECT and RLD domain containing E3 ubiquitin protein ligase 5), *PDK1* (Pyruvate Dehydrogenase Kinase 1), and *DHX58* (DExH-box helicase 58)—were consistently downregulated by both PVR overexpression and IL-10 stimulation, suggesting convergence on shared immunosuppressive targets. Pathway enrichment analysis of these shared genes revealed significant involvement in antiviral defense responses and cytokine-mediated signaling in the immune system, supporting a potential role for both IL-10 and PVR in dampening proinflammatory and antiviral transcriptional programs in T cells.

**TABLE 11:** PVR overexpression in CD4^+^ T cells partially recapitulates IL-10–mediated immunosuppressive transcriptional programs across multiple immune cell types.

IL-10 Related datasets		CD4 T + Cells stimulated with CD155 expressing L cells) (GSE194293)	
		Upregulated genes (113)	Downregulated genes (938)
CD4 + T cell stimulated with IL-10 (GSE17199)	Upregulated (436)	0	21
	Downregulated (355)	6	9
CD4 + T cell stimulated with IL-10 (GSE198963)	Upregulated (191)	1	28
	Downregulated (77)	2	8
Peripheral blood mononuclear cells derived monocytes treated with IL10 (GSE43700)	Upregulated (1,313)	4	78
	Downregulated (1,641)	15	77
Human monocyte derived macrophages stimulated with LPS and IL-10 blocking antibody (GSE181250)	Upregulated (346)	5	39
	Downregulated (330)	5	24
Monocyte derived DC cell stimulated with IL-10 (GSE180761)	Upregulated (1,872)	14	111
	Downregulated (1,222)	23	57

Comparative transcriptomic analyses reveal both distinct and overlapping gene expression profiles between PVR-overexpressing CD4^+^ T cells (GSE194293) and IL-10–stimulated CD4^+^ T cells (GSE17199), with limited global convergence but shared downregulation of antiviral and cytokine-associated genes. Comparison with an independent IL-10–treated CD4^+^ T cell dataset (GSE198963) demonstrates minimal overlap among upregulated genes, yet significant convergence among downregulated immunoregulatory genes. Cross-cell-type analyses show that PVR overexpression in CD4^+^ T cells overlap with IL-10–regulated transcriptional programs in monocytes (GSE43700), including bidirectional modulation of interferon signaling pathways. Genes downregulated by PVR overexpression are further enriched among IL-10–inhibited and IL-10–induced gene sets in monocyte-derived macrophages (GSE181250), supporting functional convergence in suppressing inflammatory signaling and cell migration. Shared transcriptional signatures between PVR-overexpressing CD4^+^ T cells and IL-10–regulated dendritic cells (GSE180761) further indicate conserved immunosuppressive pathway engagement involving cytokine signaling and ERK regulation.

We extended our analysis by examining a second IL-10 treated CD4^+^ T cells (GSE198963) to compare gene expression overlaps with PVR-overexpressing CD4^+^ T cells. Only a single gene, *SLC27A2*, was found to be upregulated by both PVR and IL-10. In contrast, 28 genes downregulated by PVR overlapped with genes upregulated by IL-10, and eight genes—*TAGAP-AS1 (TAGAP antisense RNA 1)*, *PCSK6* (proprotein convertase subtilisin/Kexin type 6), *TAGAP* (T cell activation RhoGTPase activating protein), *BHLHA15* (basic helix-loop-helix family member A15), *RHOB* (Ras homolog family member B)*, PKD1L3* (polycystin 1 like 3, transient receptor potential channel interacting), *SESN2* (Sestrin 2), and *N4BP3* (NEDD4 binding protein 3) were downregulated by both PVR and IL-10, further supporting a partially shared immunosuppressive transcriptional profile.

To investigate this relationship in innate immune cells, we compared the transcriptomic data from PVR-overexpressing CD4^+^ T cells with IL-10-stimulated monocytes (GSE43700). Among the overlapping genes, four were upregulated by both PVR and IL-10, while 15 were upregulated by PVR but downregulated by IL-10. Strikingly, 78 genes downregulated by PVR overlapped with genes upregulated by IL-10, and 77 PVR-downregulated genes also overlapped with IL-10-downregulated genes. These bidirectional overlaps suggest that PVR and IL-10 may regulate a common set of immunoregulatory genes, either in parallel or in opposing directions depending on the cellular context. Pathway enrichment analysis of the 77 overlapping genes highlighted involvement in interferon alpha/beta signaling, modulation of host response by IFN-stimulated genes, and other immune regulatory pathways. Further supporting the idea of functional convergence, monocyte derived macrophages stimulated with LPS and IL-10 blocking antibody (GSE181250) shows that 39 genes downregulated by PVR in CD4^+^ T cells were also upregulated upon IL-10 inhibition, suggesting shared immunosuppressive targets between PVR overexpression in T cells and IL-10 stimulated monocytes. These genes were enriched in pathways including inflammatory bowel disease signaling, positive regulation of cell migration, regulation of cell-cell adhesion, Fas ligand pathway and stress induction of heat shock proteins, overview of proinflammatory and profibrotic mediators, and other inflammation pathways (Supplementary Figure 3D). Conversely, 24 genes downregulated by PVR also overlapped with genes downregulated upon IL-10 blockade (IL-10 promoted genes), further reinforcing a shared immunosuppressive transcriptional axis between PVR and IL-10. Finally, monocyte derived DC cell stimulated with IL-10 (GSE180761) demonstrates substantial overlaps between PVR-downregulated genes in CD4^+^ T cells and IL-10-regulated genes in DCs: 57 PVR-downregulated genes overlapped with IL-10-downregulated genes, while 111 overlapped with IL-10-upregulated genes. Pathway enrichment analysis of the 57 overlapped downregulated genes indicated associations with pathways such as positive regulation of monoatomic ion transport, neovascularization process, regulation of extracellular signal-regulated kinase 1 (ERK1) and ERK2 cascade (Supplementary Figure 3E).

Taken together, these results indicate that PVR overexpression in CD4^+^ T cells share a significant number of immunosuppressive genes and signaling pathways with IL-10–mediated responses across multiple immune cell types—including CD4^+^ T cells, monocytes, and dendritic cells. While substantial transcriptional overlap suggests a shared functional program, distinct gene expression signatures also emerge, underscoring that PVR and IL-10 regulate both overlapping and unique components of immune suppression.

## 4 Discussion

In this study, we present a comprehensive transcriptomic analysis that delineates the molecular and cellular mechanisms by which COPD drives lung inflammation, injury, and immune dysregulation: Our findings reveal several major themes: (1) COPD promotes the upregulation of cytokines, secretomic proteins, plasma membrane proteins, CD markers, innate immune genes, and trained immunity genes. Notably, the suppression of the trained immunity promoter SET7 and overexpression of IL-37 reduces the expression of 58 and 12 COPD-upregulated genes, respectively, highlighting their potential as anti-inflammatory regulators. (2) COPD-induced lung injury is associated with the transcriptional upregulation of genes across eight distinct forms of cell death, including autosis, autophagy, parthanatos, immunogenic cell death, mitochondrial permeability transition cell death, lysosomal cell death, mitotic cell death, and proliferation-associated cell death, in addition to the four classical cell death pathways—including pyroptosis, apoptosis, necroptosis, and ferroptosis, reflecting widespread cellular stress and damage. (3) COPD significantly alters the transcriptional landscape in 10 out of 14 human lung cell types. Differentially expressed genes were upregulated in respiratory cilliated cells, alveolar cells type I, B cells, fibroblasts, mast cells, T cells, and plasma cell. Importantly, we observed pathological trans-differentiation, with upregulation of 52 cell-type-specific markers, including 18 markers with expression exceeding 5% of total cell-type-specific marker genes —indicating broad shifts in cell identity and function. (4) COPD upregulate genes associated with EMT and fibrosis. These changes suggest sustained activation of proinflammatory, profibrotic, and remodeling pathways. (5) In alveolar macrophages from smokers with COPD, we observed significant downregulation of 11 inhibitory, 7 stimulatory, and one dual-function immune checkpoint ligands, indicating broad dysregulation of immune checkpoint signaling. (6) The inhibitory immune checkpoint ligand PVR (CD155) was significantly downregulated in alveolar macrophages from patients with severe emphysema and COPD, suggesting impaired immunosuppressive signaling in the lung microenvironment. (7) Functional analysis showed that overexpression of PVR in alveolar and interstitial macrophages suppressed the expression of 12 genes —including *IL-1RL1*, *MUC1, CLDN1, IL1A, VEGFA, OAS1, CCL20, IL-13, IFIH1, IRF7, MX1*, and *SERPINA1—*highlighting its potential role in regulating macrophage-mediated inflammation in COPD. (8) We found significant transcriptional reprogramming of Tregs in COPD, with downregulation of 74 Treg-related genes across multiple gene sets, including six from the HPA, 55 smoke-sensitive genes, five FoxP3^+^ Treg genes, four LPS-responsive lung Treg genes, and 5 TIGIT^+^ Treg genes, these changes indicate a weakening of Treg-mediated immune tolerance. (9) COPD lungs showed downregulation of 31 genes normally upregulated by IL-10, suggesting impaired IL-10–mediated anti-inflammatory signaling in the COPD lung environment. (10) Among 190 genes downregulated by PVR overexpression in CD4^+^ T cells, a substantial number overlapped with IL-10–inhibited genes in CD4^+^ T cells and monocytes. This convergence suggests that PVR and IL-10 may operate through overlapping immunosuppressive pathways, which are disrupted in COPD.

Our additional analyses (Supplementary Figure 4) demonstrate that COPD is associated with a consistent reduction in the expression of key Treg immunosuppressive genes across both human disease samples and murine COPD models. By presenting differential expression data for canonical Treg markers, including FOXP3, TIGIT, and IL-10, we show that the observed suppression reflects a reproducible transcriptional trend rather than isolated gene-specific effects. These findings support the concept that chronic inflammatory conditions in COPD are accompanied by impaired Treg-mediated immune regulation, which may contribute to sustained immune activation and disease progression. Importantly, to further strengthen and extend these observations, we are currently performing RNA sequencing on lung tissues from COPD and cigarette smoke–exposed samples. Upon completion, these datasets will enable more refined transcriptomic analyses and facilitate experimental validation of Treg-associated gene dysregulation, providing an independent confirmation of our findings and allowing deeper mechanistic interrogation in future studies.

**FIGURE 4:**
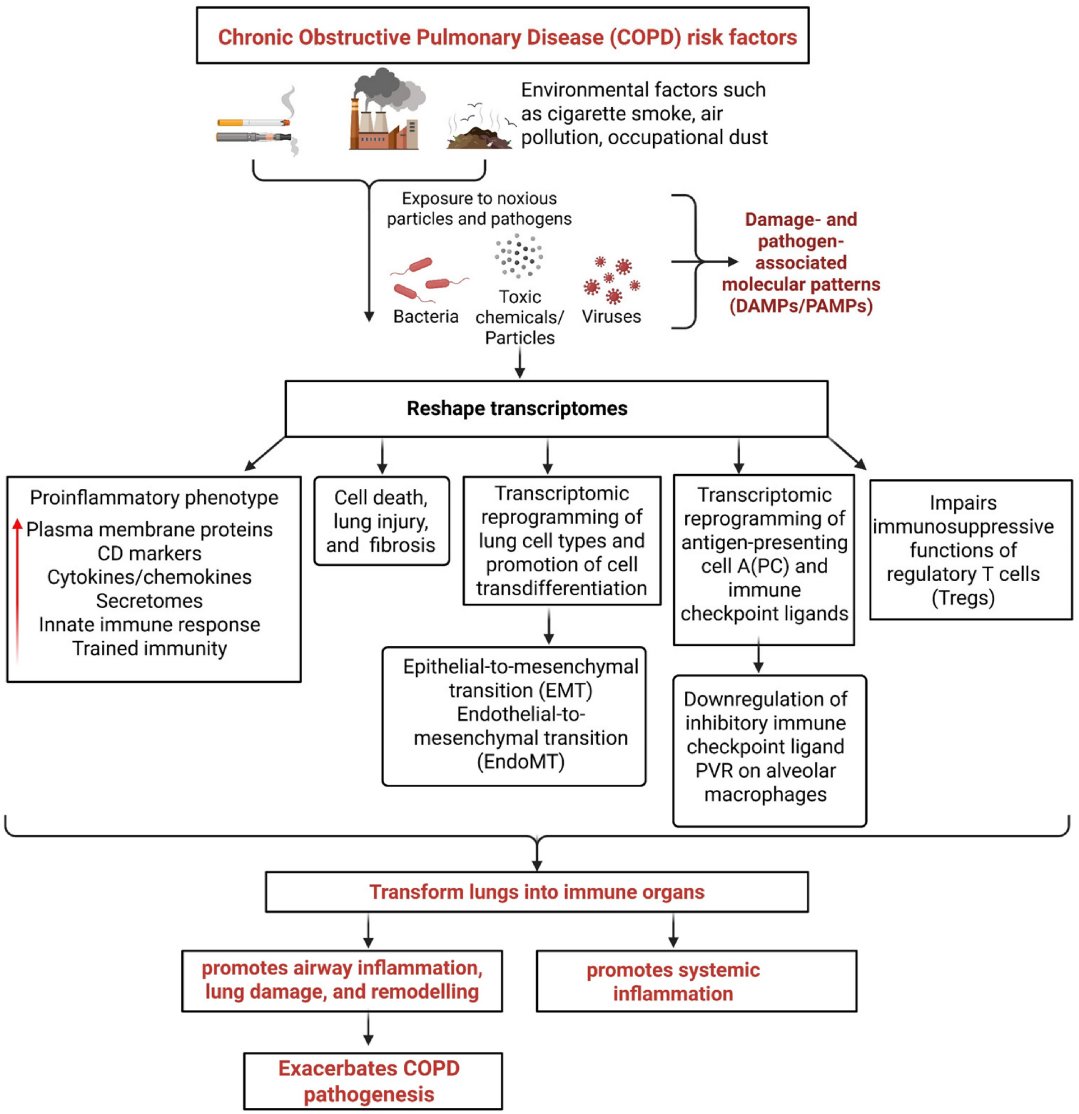
Proposed working model and summary of findings.

Our data support a model (Figure 4) in which environmental and microbial stimuli—such as cigarette smoke, air pollution, and DAMPs/PAMPs—drive widespread transcriptomic reprogramming in the lung. These changes include the upregulation of genes encoding plasma membrane proteins (e.g., CD markers), cytokines, chemokines, and secretomes. This dysregulated gene expression landscape promotes multiple forms of cell death, fibrotic remodeling, and pathological trans-differentiation of resident lung cell types. We also identified transcriptomic changes associated with antigen presentation dysfunction (APD) and altered expression of immune checkpoint ligands and receptors. Additionally, our findings indicate a compromised immunosuppressive function of regulatory T cells (Tregs) within the COPD lung microenvironment.

Although numerous studies have described cellular and inflammatory alterations in COPD, how EMT–associated transcriptional programs operate within specific cellular contexts to drive disease progression remains incompletely understood. In this study, our goal was not to assign causality at the single-cell or functional level, but rather to perform a transcriptomic, hypothesis-generating analysis aimed at identifying reproducible and biologically grounded EMT signatures associated with COPD. Importantly, the EMT gene sets analyzed here were not arbitrarily selected; they comprise well-established, experimentally validated EMT-associated genes derived from extensive prior research. Leveraging these curated gene lists enables a knowledge-driven transcriptomic framework that complements, rather than replaces, cell-resolved approaches and differs conceptually from automated pathway tools by anchoring interpretation in established biology. Consistent with prior reports demonstrating a contributory role for EMT in COPD pathogenesis (11–13, 78), our findings provide additional mechanistic hypotheses by highlighting coordinated EMT-related transcriptional changes at the tissue level. These data should therefore be interpreted as candidate molecular programs that are likely to reflect altered epithelial plasticity and remodeling in COPD, which will require validation through spatially resolved transcriptomics and targeted functional assays. Such studies, which we plan to pursue pending additional funding, will be essential to define how EMT-associated pathways function within discrete lung cell populations to promote structural remodeling and disease progression.

Together, these changes suggest that the COPD lung adopts characteristics of an immune-endocrine organ, sustaining chronic airway inflammation, promoting tissue destruction and remodeling, and ultimately driving disease progression. Furthermore, these local changes may contribute to systemic inflammation observed in COPD patients, underscoring the multisystemic impact of the disease.

In summary, our integrative transcriptomic analysis reveals that COPD orchestrates a complex network of cellular and molecular events involving immune activation, epithelial and endothelial remodeling, induction of diverse cell death programs, and disruption of key immunoregulatory mechanisms. Notably, we identify a novel convergence between the inhibitory checkpoint molecule PVR and the anti-inflammatory cytokine IL-10, both of which are downregulated in COPD and share overlapping immunosuppressive gene targets. These findings suggest that the loss of PVR and IL-10–mediated signaling contributes to the chronic inflammation and immune dysregulation characteristic of COPD. Moreover, the observed transcriptional reprogramming of regulatory T cells, professional antigen-presenting cells, and structural lung cells underscores the systemic nature of COPD pathogenesis. Collectively, this study provides a comprehensive molecular framework for understanding COPD progression and identifies potential therapeutic targets—such as SET7, IL-37, PVR, and IL-10—for restoring immune balance and limiting lung damage in COPD patients.

## Data Availability

The data analyzed in this study are publicly available and were obtained from the NIH Gene Expression Omnibus (GEO) repository. The accession numbers for all datasets used are provided in the manuscript. No new datasets were generated in this study.

## Footnotes

1
https://www.ncbi.nlm.nih.gov/gds

2
https://www.ncbi.nlm.nih.gov/geo/geo2r/

3
https://metascape.org/gp/index.html#/main/step1

4
https://www.who.int/news/item/15-11-2023-smoking-is-the-leading-cause-of-chronic-obstructive-pulmonary-disease
