# Supplementary material for: Chronic obstructive pulmonary disease reprograms the lung into an immune organ through trained immunity, cell death networks, and immune checkpoint dysregulation
Source: Front Med (Lausanne). 2026 Jan 28;13:1721780. doi: 10.3389/fmed.2026.1721780 (PMC12891065; doi:10.3389/fmed.2026.1721780)
Supplement: Supplementary file 1 [file Presentation_1.PPTX]

## Slide 1
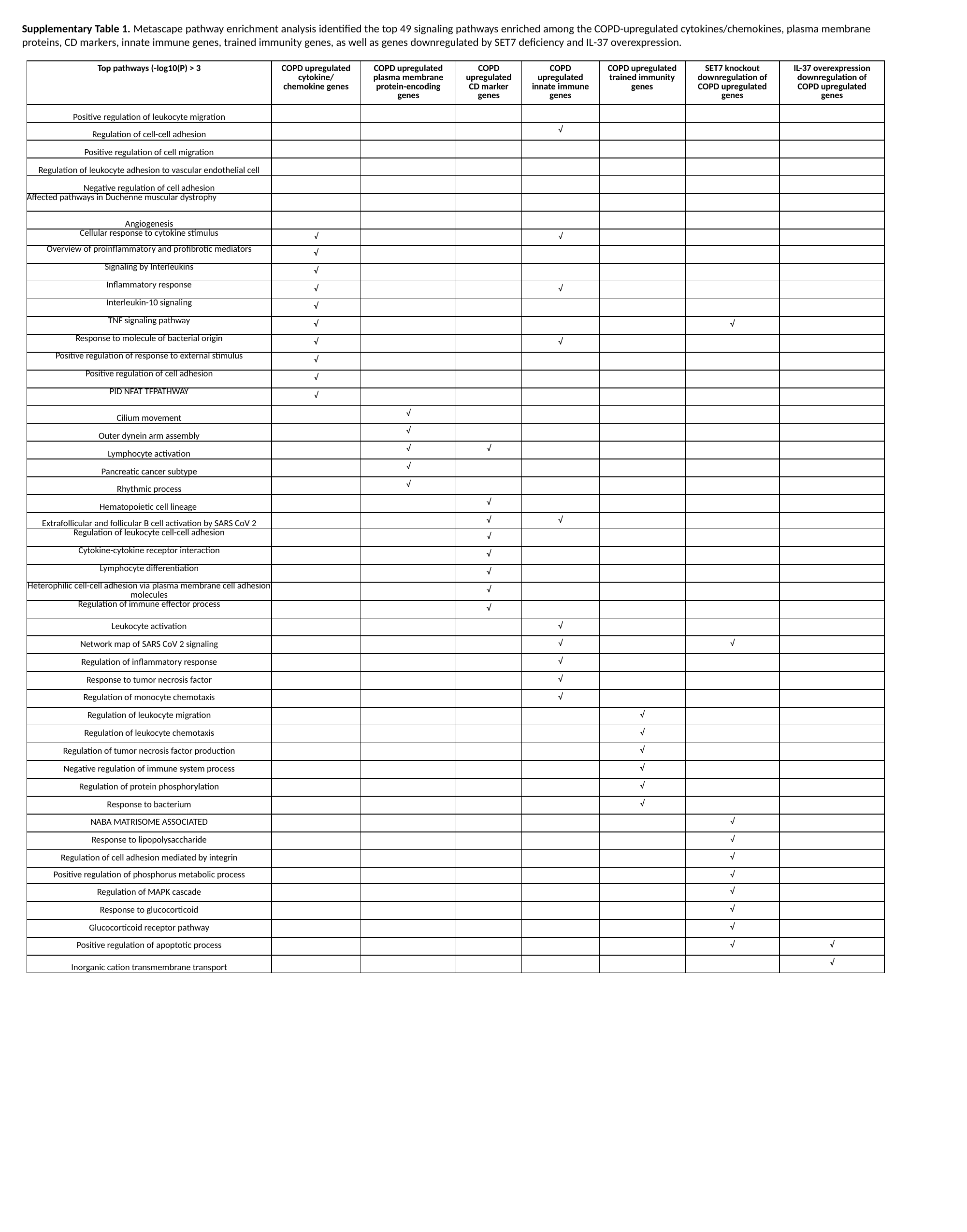

Supplementary Table 1. Metascape pathway enrichment analysis identified the top 49 signaling pathways enriched among the COPD-upregulated cytokines/chemokines, plasma membrane proteins, CD markers, innate immune genes, trained immunity genes, as well as genes downregulated by SET7 deficiency and IL-37 overexpression.
| Top pathways (-log10(P) > 3 | COPD upregulated cytokine/ chemokine genes | COPD upregulated plasma membrane protein-encoding genes | COPD upregulated CD marker genes | COPD upregulated innate immune genes | COPD upregulated trained immunity genes | SET7 knockout downregulation of COPD upregulated genes | IL-37 overexpression downregulation of COPD upregulated genes |
| --- | --- | --- | --- | --- | --- | --- | --- |
| Positive regulation of leukocyte migration | | | | | | | |
| Regulation of cell-cell adhesion | | | | √ | | | |
| Positive regulation of cell migration | | | | | | | |
| Regulation of leukocyte adhesion to vascular endothelial cell | | | | | | | |
| Negative regulation of cell adhesion | | | | | | | |
| Affected pathways in Duchenne muscular dystrophy | | | | | | | |
| Angiogenesis | | | | | | | |
| Cellular response to cytokine stimulus | √ | | | √ | | | |
| Overview of proinflammatory and profibrotic mediators | √ | | | | | | |
| Signaling by Interleukins | √ | | | | | | |
| Inflammatory response | √ | | | √ | | | |
| Interleukin-10 signaling | √ | | | | | | |
| TNF signaling pathway | √ | | | | | √ | |
| Response to molecule of bacterial origin | √ | | | √ | | | |
| Positive regulation of response to external stimulus | √ | | | | | | |
| Positive regulation of cell adhesion | √ | | | | | | |
| PID NFAT TFPATHWAY | √ | | | | | | |
| Cilium movement | | √ | | | | | |
| Outer dynein arm assembly | | √ | | | | | |
| Lymphocyte activation | | √ | √ | | | | |
| Pancreatic cancer subtype | | √ | | | | | |
| Rhythmic process | | √ | | | | | |
| Hematopoietic cell lineage | | | √ | | | | |
| Extrafollicular and follicular B cell activation by SARS CoV 2 | | | √ | √ | | | |
| Regulation of leukocyte cell-cell adhesion | | | √ | | | | |
| Cytokine-cytokine receptor interaction | | | √ | | | | |
| Lymphocyte differentiation | | | √ | | | | |
| Heterophilic cell-cell adhesion via plasma membrane cell adhesion molecules | | | √ | | | | |
| Regulation of immune effector process | | | √ | | | | |
| Leukocyte activation | | | | √ | | | |
| Network map of SARS CoV 2 signaling | | | | √ | | √ | |
| Regulation of inflammatory response | | | | √ | | | |
| Response to tumor necrosis factor | | | | √ | | | |
| Regulation of monocyte chemotaxis | | | | √ | | | |
| Regulation of leukocyte migration | | | | | √ | | |
| Regulation of leukocyte chemotaxis | | | | | √ | | |
| Regulation of tumor necrosis factor production | | | | | √ | | |
| Negative regulation of immune system process | | | | | √ | | |
| Regulation of protein phosphorylation | | | | | √ | | |
| Response to bacterium | | | | | √ | | |
| NABA MATRISOME ASSOCIATED | | | | | | √ | |
| Response to lipopolysaccharide | | | | | | √ | |
| Regulation of cell adhesion mediated by integrin | | | | | | √ | |
| Positive regulation of phosphorus metabolic process | | | | | | √ | |
| Regulation of MAPK cascade | | | | | | √ | |
| Response to glucocorticoid | | | | | | √ | |
| Glucocorticoid receptor pathway | | | | | | √ | |
| Positive regulation of apoptotic process | | | | | | √ | √ |
| Inorganic cation transmembrane transport | | | | | | | √ |

## Slide 2
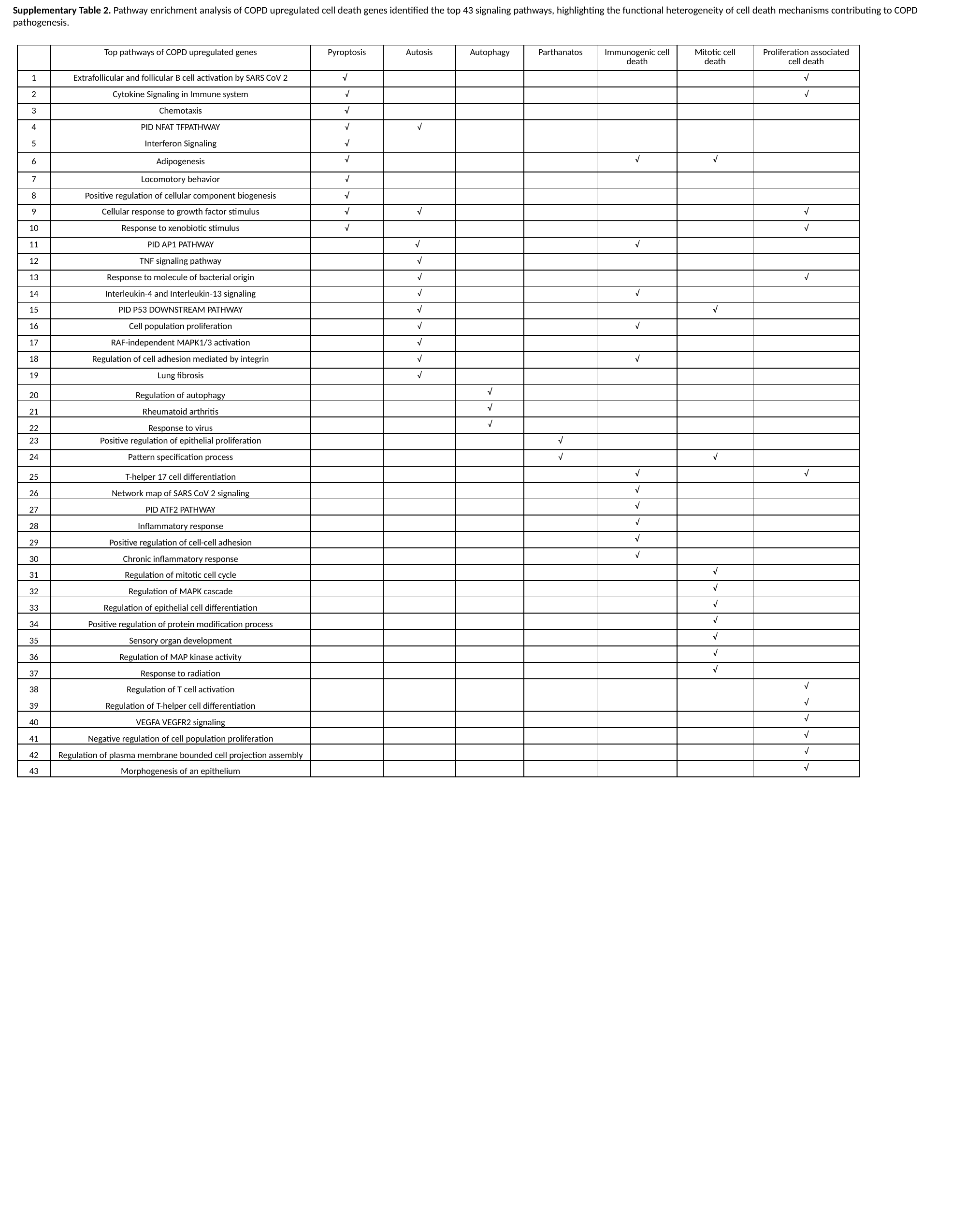

Supplementary Table 2. Pathway enrichment analysis of COPD upregulated cell death genes identified the top 43 signaling pathways, highlighting the functional heterogeneity of cell death mechanisms contributing to COPD pathogenesis.
| | Top pathways of COPD upregulated genes | Pyroptosis | Autosis | Autophagy | Parthanatos | Immunogenic cell death | Mitotic cell death | Proliferation associated cell death |
| --- | --- | --- | --- | --- | --- | --- | --- | --- |
| 1 | Extrafollicular and follicular B cell activation by SARS CoV 2 | √ | | | | | | √ |
| 2 | Cytokine Signaling in Immune system | √ | | | | | | √ |
| 3 | Chemotaxis | √ | | | | | | |
| 4 | PID NFAT TFPATHWAY | √ | √ | | | | | |
| 5 | Interferon Signaling | √ | | | | | | |
| 6 | Adipogenesis | √ | | | | √ | √ | |
| 7 | Locomotory behavior | √ | | | | | | |
| 8 | Positive regulation of cellular component biogenesis | √ | | | | | | |
| 9 | Cellular response to growth factor stimulus | √ | √ | | | | | √ |
| 10 | Response to xenobiotic stimulus | √ | | | | | | √ |
| 11 | PID AP1 PATHWAY | | √ | | | √ | | |
| 12 | TNF signaling pathway | | √ | | | | | |
| 13 | Response to molecule of bacterial origin | | √ | | | | | √ |
| 14 | Interleukin-4 and Interleukin-13 signaling | | √ | | | √ | | |
| 15 | PID P53 DOWNSTREAM PATHWAY | | √ | | | | √ | |
| 16 | Cell population proliferation | | √ | | | √ | | |
| 17 | RAF-independent MAPK1/3 activation | | √ | | | | | |
| 18 | Regulation of cell adhesion mediated by integrin | | √ | | | √ | | |
| 19 | Lung fibrosis | | √ | | | | | |
| 20 | Regulation of autophagy | | | √ | | | | |
| 21 | Rheumatoid arthritis | | | √ | | | | |
| 22 | Response to virus | | | √ | | | | |
| 23 | Positive regulation of epithelial proliferation | | | | √ | | | |
| 24 | Pattern specification process | | | | √ | | √ | |
| 25 | T-helper 17 cell differentiation | | | | | √ | | √ |
| 26 | Network map of SARS CoV 2 signaling | | | | | √ | | |
| 27 | PID ATF2 PATHWAY | | | | | √ | | |
| 28 | Inflammatory response | | | | | √ | | |
| 29 | Positive regulation of cell-cell adhesion | | | | | √ | | |
| 30 | Chronic inflammatory response | | | | | √ | | |
| 31 | Regulation of mitotic cell cycle | | | | | | √ | |
| 32 | Regulation of MAPK cascade | | | | | | √ | |
| 33 | Regulation of epithelial cell differentiation | | | | | | √ | |
| 34 | Positive regulation of protein modification process | | | | | | √ | |
| 35 | Sensory organ development | | | | | | √ | |
| 36 | Regulation of MAP kinase activity | | | | | | √ | |
| 37 | Response to radiation | | | | | | √ | |
| 38 | Regulation of T cell activation | | | | | | | √ |
| 39 | Regulation of T-helper cell differentiation | | | | | | | √ |
| 40 | VEGFA VEGFR2 signaling | | | | | | | √ |
| 41 | Negative regulation of cell population proliferation | | | | | | | √ |
| 42 | Regulation of plasma membrane bounded cell projection assembly | | | | | | | √ |
| 43 | Morphogenesis of an epithelium | | | | | | | √ |

## Slide 3
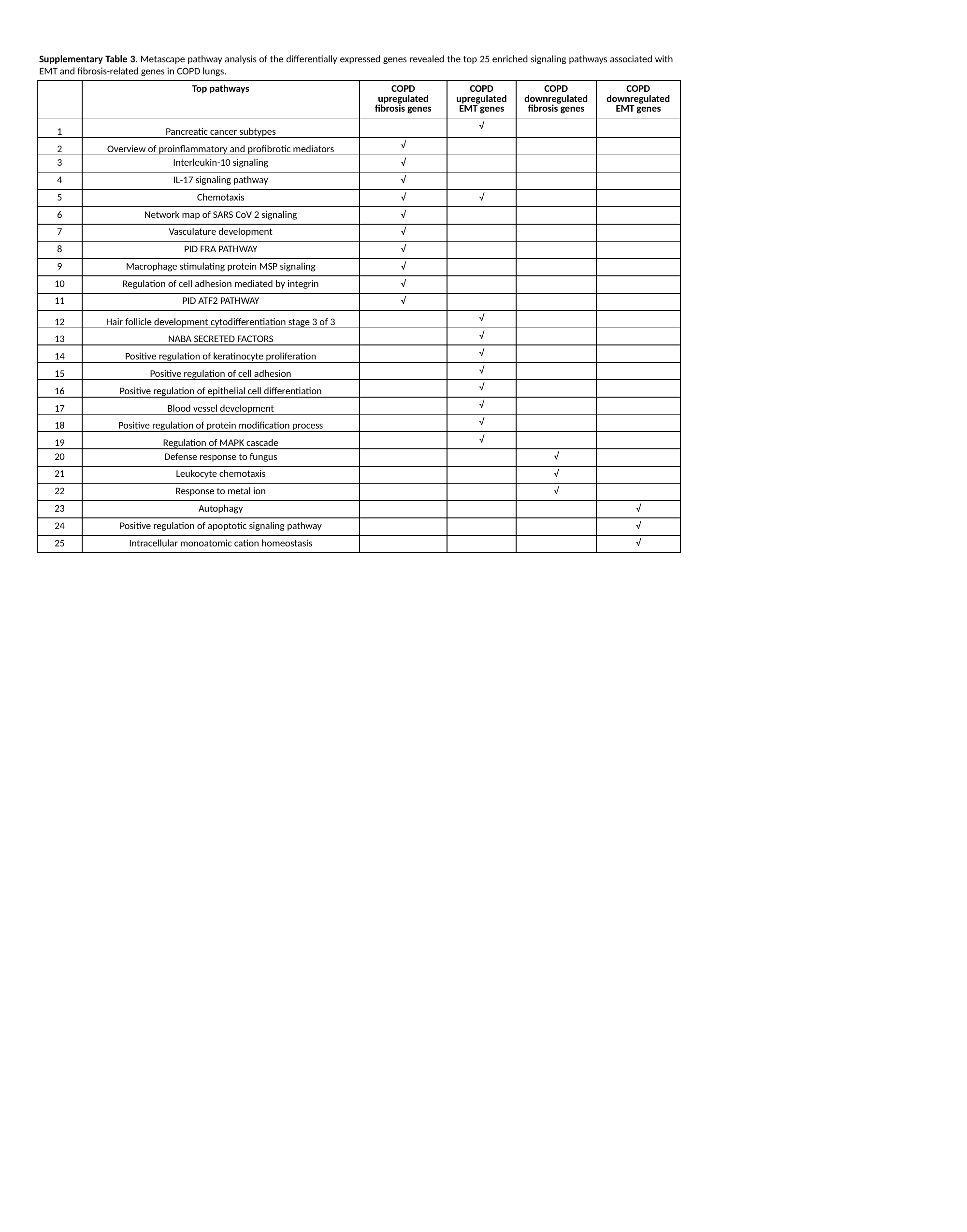

Supplementary Table 3. Metascape pathway analysis of the differentially expressed genes revealed the top 25 enriched signaling pathways associated with EMT and fibrosis-related genes in COPD lungs.
| | Top pathways | COPD upregulated fibrosis genes | COPD upregulated EMT genes | COPD downregulated fibrosis genes | COPD downregulated EMT genes |
| --- | --- | --- | --- | --- | --- |
| 1 | Pancreatic cancer subtypes | | √ | | |
| 2 | Overview of proinflammatory and profibrotic mediators | √ | | | |
| 3 | Interleukin-10 signaling | √ | | | |
| 4 | IL-17 signaling pathway | √ | | | |
| 5 | Chemotaxis | √ | √ | | |
| 6 | Network map of SARS CoV 2 signaling | √ | | | |
| 7 | Vasculature development | √ | | | |
| 8 | PID FRA PATHWAY | √ | | | |
| 9 | Macrophage stimulating protein MSP signaling | √ | | | |
| 10 | Regulation of cell adhesion mediated by integrin | √ | | | |
| 11 | PID ATF2 PATHWAY | √ | | | |
| 12 | Hair follicle development cytodifferentiation stage 3 of 3 | | √ | | |
| 13 | NABA SECRETED FACTORS | | √ | | |
| 14 | Positive regulation of keratinocyte proliferation | | √ | | |
| 15 | Positive regulation of cell adhesion | | √ | | |
| 16 | Positive regulation of epithelial cell differentiation | | √ | | |
| 17 | Blood vessel development | | √ | | |
| 18 | Positive regulation of protein modification process | | √ | | |
| 19 | Regulation of MAPK cascade | | √ | | |
| 20 | Defense response to fungus | | | √ | |
| 21 | Leukocyte chemotaxis | | | √ | |
| 22 | Response to metal ion | | | √ | |
| 23 | Autophagy | | | | √ |
| 24 | Positive regulation of apoptotic signaling pathway | | | | √ |
| 25 | Intracellular monoatomic cation homeostasis | | | | √ |

## Slide 4
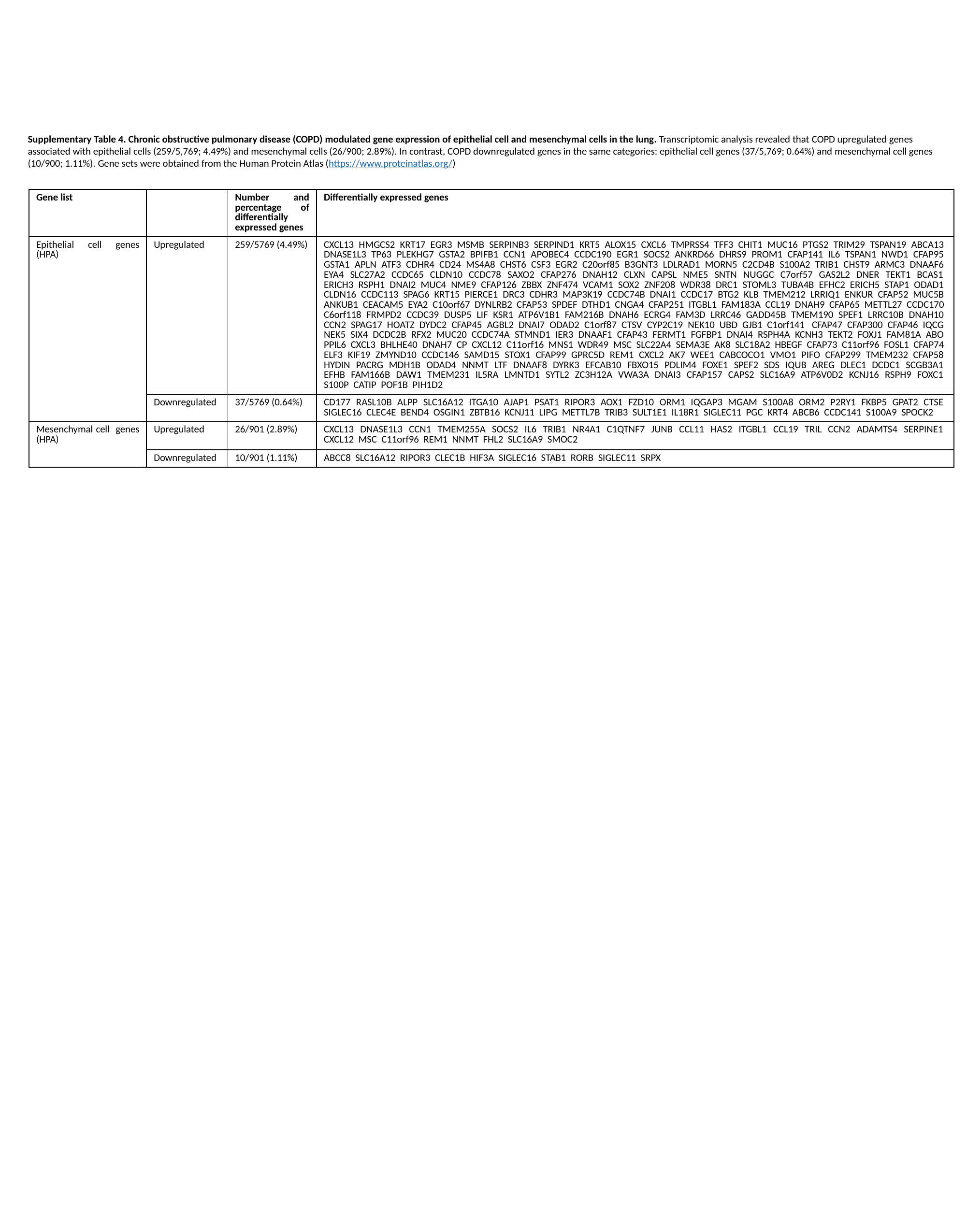

Supplementary Table 4. Chronic obstructive pulmonary disease (COPD) modulated gene expression of epithelial cell and mesenchymal cells in the lung. Transcriptomic analysis revealed that COPD upregulated genes associated with epithelial cells (259/5,769; 4.49%) and mesenchymal cells (26/900; 2.89%). In contrast, COPD downregulated genes in the same categories: epithelial cell genes (37/5,769; 0.64%) and mesenchymal cell genes (10/900; 1.11%). Gene sets were obtained from the Human Protein Atlas (https://www.proteinatlas.org/)
| Gene list | | Number and percentage of differentially expressed genes | Differentially expressed genes |
| --- | --- | --- | --- |
| Epithelial cell genes (HPA) | Upregulated | 259/5769 (4.49%) | CXCL13 HMGCS2 KRT17 EGR3 MSMB SERPINB3 SERPIND1 KRT5 ALOX15 CXCL6 TMPRSS4 TFF3 CHIT1 MUC16 PTGS2 TRIM29 TSPAN19 ABCA13 DNASE1L3 TP63 PLEKHG7 GSTA2 BPIFB1 CCN1 APOBEC4 CCDC190 EGR1 SOCS2 ANKRD66 DHRS9 PROM1 CFAP141 IL6 TSPAN1 NWD1 CFAP95 GSTA1 APLN ATF3 CDHR4 CD24 MS4A8 CHST6 CSF3 EGR2 C20orf85 B3GNT3 LDLRAD1 MORN5 C2CD4B S100A2 TRIB1 CHST9 ARMC3 DNAAF6 EYA4 SLC27A2 CCDC65 CLDN10 CCDC78 SAXO2 CFAP276 DNAH12 CLXN CAPSL NME5 SNTN NUGGC C7orf57 GAS2L2 DNER TEKT1 BCAS1 ERICH3 RSPH1 DNAI2 MUC4 NME9 CFAP126 ZBBX ZNF474 VCAM1 SOX2 ZNF208 WDR38 DRC1 STOML3 TUBA4B EFHC2 ERICH5 STAP1 ODAD1 CLDN16 CCDC113 SPAG6 KRT15 PIERCE1 DRC3 CDHR3 MAP3K19 CCDC74B DNAI1 CCDC17 BTG2 KLB TMEM212 LRRIQ1 ENKUR CFAP52 MUC5B ANKUB1 CEACAM5 EYA2 C10orf67 DYNLRB2 CFAP53 SPDEF DTHD1 CNGA4 CFAP251 ITGBL1 FAM183A CCL19 DNAH9 CFAP65 METTL27 CCDC170 C6orf118 FRMPD2 CCDC39 DUSP5 LIF KSR1 ATP6V1B1 FAM216B DNAH6 ECRG4 FAM3D LRRC46 GADD45B TMEM190 SPEF1 LRRC10B DNAH10 CCN2 SPAG17 HOATZ DYDC2 CFAP45 AGBL2 DNAI7 ODAD2 C1orf87 CTSV CYP2C19 NEK10 UBD GJB1 C1orf141 CFAP47 CFAP300 CFAP46 IQCG NEK5 SIX4 DCDC2B RFX2 MUC20 CCDC74A STMND1 IER3 DNAAF1 CFAP43 FERMT1 FGFBP1 DNAI4 RSPH4A KCNH3 TEKT2 FOXJ1 FAM81A ABO PPIL6 CXCL3 BHLHE40 DNAH7 CP CXCL12 C11orf16 MNS1 WDR49 MSC SLC22A4 SEMA3E AK8 SLC18A2 HBEGF CFAP73 C11orf96 FOSL1 CFAP74 ELF3 KIF19 ZMYND10 CCDC146 SAMD15 STOX1 CFAP99 GPRC5D REM1 CXCL2 AK7 WEE1 CABCOCO1 VMO1 PIFO CFAP299 TMEM232 CFAP58 HYDIN PACRG MDH1B ODAD4 NNMT LTF DNAAF8 DYRK3 EFCAB10 FBXO15 PDLIM4 FOXE1 SPEF2 SDS IQUB AREG DLEC1 DCDC1 SCGB3A1 EFHB FAM166B DAW1 TMEM231 IL5RA LMNTD1 SYTL2 ZC3H12A VWA3A DNAI3 CFAP157 CAPS2 SLC16A9 ATP6V0D2 KCNJ16 RSPH9 FOXC1 S100P CATIP POF1B PIH1D2 |
| | Downregulated | 37/5769 (0.64%) | CD177 RASL10B ALPP SLC16A12 ITGA10 AJAP1 PSAT1 RIPOR3 AOX1 FZD10 ORM1 IQGAP3 MGAM S100A8 ORM2 P2RY1 FKBP5 GPAT2 CTSE SIGLEC16 CLEC4E BEND4 OSGIN1 ZBTB16 KCNJ11 LIPG METTL7B TRIB3 SULT1E1 IL18R1 SIGLEC11 PGC KRT4 ABCB6 CCDC141 S100A9 SPOCK2 |
| Mesenchymal cell genes (HPA) | Upregulated | 26/901 (2.89%) | CXCL13 DNASE1L3 CCN1 TMEM255A SOCS2 IL6 TRIB1 NR4A1 C1QTNF7 JUNB CCL11 HAS2 ITGBL1 CCL19 TRIL CCN2 ADAMTS4 SERPINE1 CXCL12 MSC C11orf96 REM1 NNMT FHL2 SLC16A9 SMOC2 |
| | Downregulated | 10/901 (1.11%) | ABCC8 SLC16A12 RIPOR3 CLEC1B HIF3A SIGLEC16 STAB1 RORB SIGLEC11 SRPX |

## Slide 5
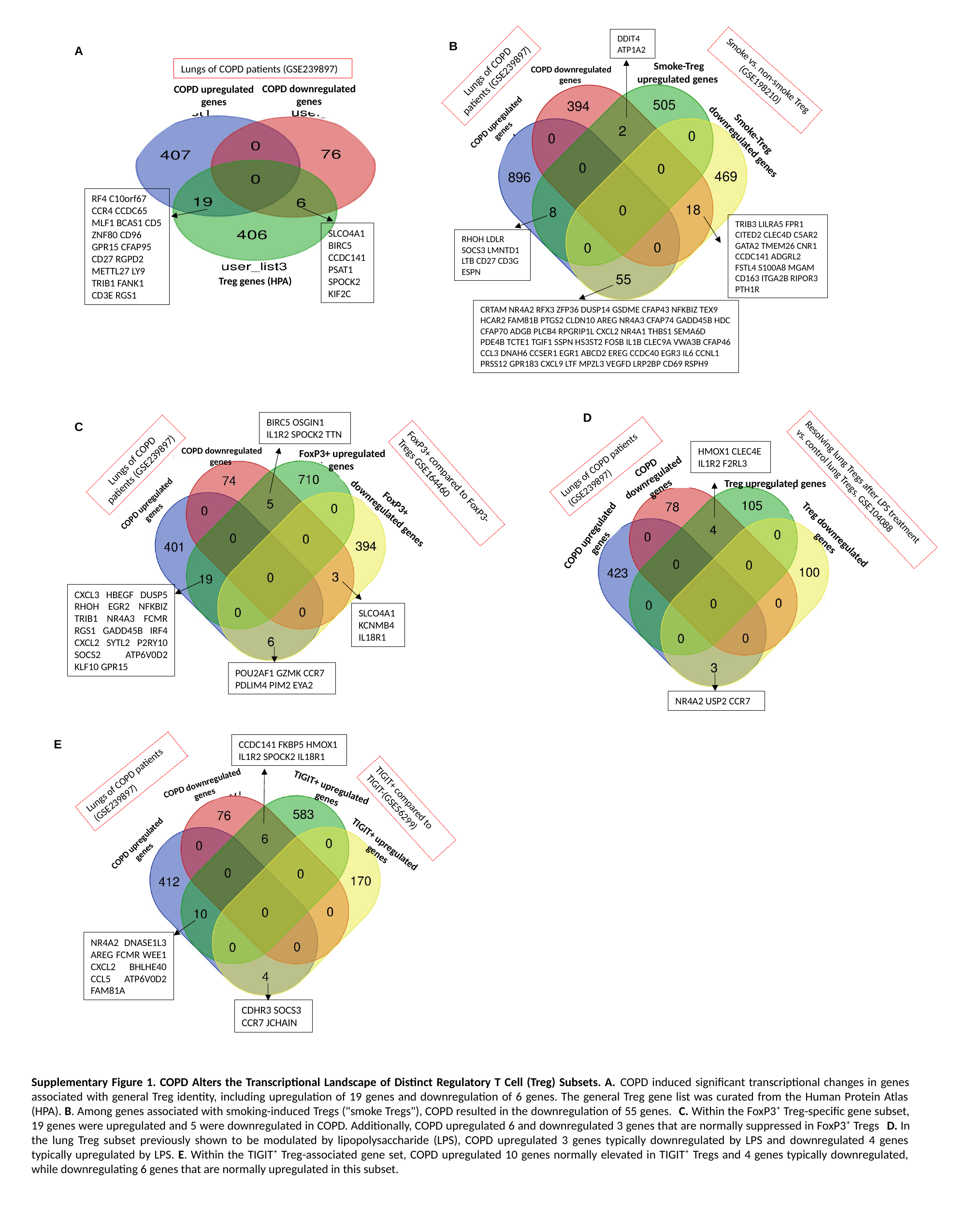

DDIT4 ATP1A2
B
A
Smoke-Treg upregulated genes
Lungs of COPD patients (GSE239897)
Lungs of COPD patients (GSE239897)
COPD downregulated genes
Smoke vs. non-smoke Treg (GSE198210)
COPD downregulated genes
COPD upregulated genes
RF4 C10orf67 CCR4 CCDC65 MLF1 BCAS1 CD5 ZNF80 CD96 GPR15 CFAP95 CD27 RGPD2 METTL27 LY9 TRIB1 FANK1 CD3E RGS1
Treg genes (HPA)
SLCO4A1 BIRC5 CCDC141 PSAT1 SPOCK2 KIF2C
COPD upregulated genes
Smoke-Treg downregulated genes
TRIB3 LILRA5 FPR1 CITED2 CLEC4D C5AR2 GATA2 TMEM26 CNR1 CCDC141 ADGRL2 FSTL4 S100A8 MGAM CD163 ITGA2B RIPOR3 PTH1R
RHOH LDLR SOCS3 LMNTD1 LTB CD27 CD3G ESPN
CRTAM NR4A2 RFX3 ZFP36 DUSP14 GSDME CFAP43 NFKBIZ TEX9 HCAR2 FAM81B PTGS2 CLDN10 AREG NR4A3 CFAP74 GADD45B HDC CFAP70 ADGB PLCB4 RPGRIP1L CXCL2 NR4A1 THBS1 SEMA6D PDE4B TCTE1 TGIF1 SSPN HS3ST2 FOSB IL1B CLEC9A VWA3B CFAP46 CCL3 DNAH6 CCSER1 EGR1 ABCD2 EREG CCDC40 EGR3 IL6 CCNL1 PRSS12 GPR183 CXCL9 LTF MPZL3 VEGFD LRP2BP CD69 RSPH9
HMOX1 CLEC4E IL1R2 F2RL3
Resolving lung Tregs after LPS treatment vs. control lung Tregs, GSE104088
NR4A2 USP2 CCR7
D
Lungs of COPD patients (GSE239897)
COPD downregulated genes
Treg upregulated genes
Treg downregulated genes
COPD upregulated genes
BIRC5 OSGIN1 IL1R2 SPOCK2 TTN
CXCL3 HBEGF DUSP5 RHOH EGR2 NFKBIZ TRIB1 NR4A3 FCMR RGS1 GADD45B IRF4 CXCL2 SYTL2 P2RY10 SOCS2 ATP6V0D2 KLF10 GPR15
SLCO4A1 KCNMB4 IL18R1
POU2AF1 GZMK CCR7 PDLIM4 PIM2 EYA2
FoxP3+ compared to FoxP3- Tregs GSE164460
C
COPD downregulated genes
FoxP3+ upregulated genes
Lungs of COPD patients (GSE239897)
FoxP3+ downregulated genes
COPD upregulated genes
E
CCDC141 FKBP5 HMOX1 IL1R2 SPOCK2 IL18R1
Lungs of COPD patients (GSE239897)
NR4A2 DNASE1L3 AREG FCMR WEE1 CXCL2 BHLHE40 CCL5 ATP6V0D2 FAM81A
CDHR3 SOCS3 CCR7 JCHAIN
COPD downregulated genes
TIGIT+ upregulated genes
TIGIT+ compared to TIGIT-(GSE56299)
COPD upregulated genes
TIGIT+ upregulated genes
Supplementary Figure 1. COPD Alters the Transcriptional Landscape of Distinct Regulatory T Cell (Treg) Subsets. A. COPD induced significant transcriptional changes in genes associated with general Treg identity, including upregulation of 19 genes and downregulation of 6 genes. The general Treg gene list was curated from the Human Protein Atlas (HPA). B. Among genes associated with smoking-induced Tregs ("smoke Tregs"), COPD resulted in the downregulation of 55 genes. C. Within the FoxP3⁺ Treg-specific gene subset, 19 genes were upregulated and 5 were downregulated in COPD. Additionally, COPD upregulated 6 and downregulated 3 genes that are normally suppressed in FoxP3⁺ Tregs D. In the lung Treg subset previously shown to be modulated by lipopolysaccharide (LPS), COPD upregulated 3 genes typically downregulated by LPS and downregulated 4 genes typically upregulated by LPS. E. Within the TIGIT⁺ Treg-associated gene set, COPD upregulated 10 genes normally elevated in TIGIT⁺ Tregs and 4 genes typically downregulated, while downregulating 6 genes that are normally upregulated in this subset.

## Slide 6
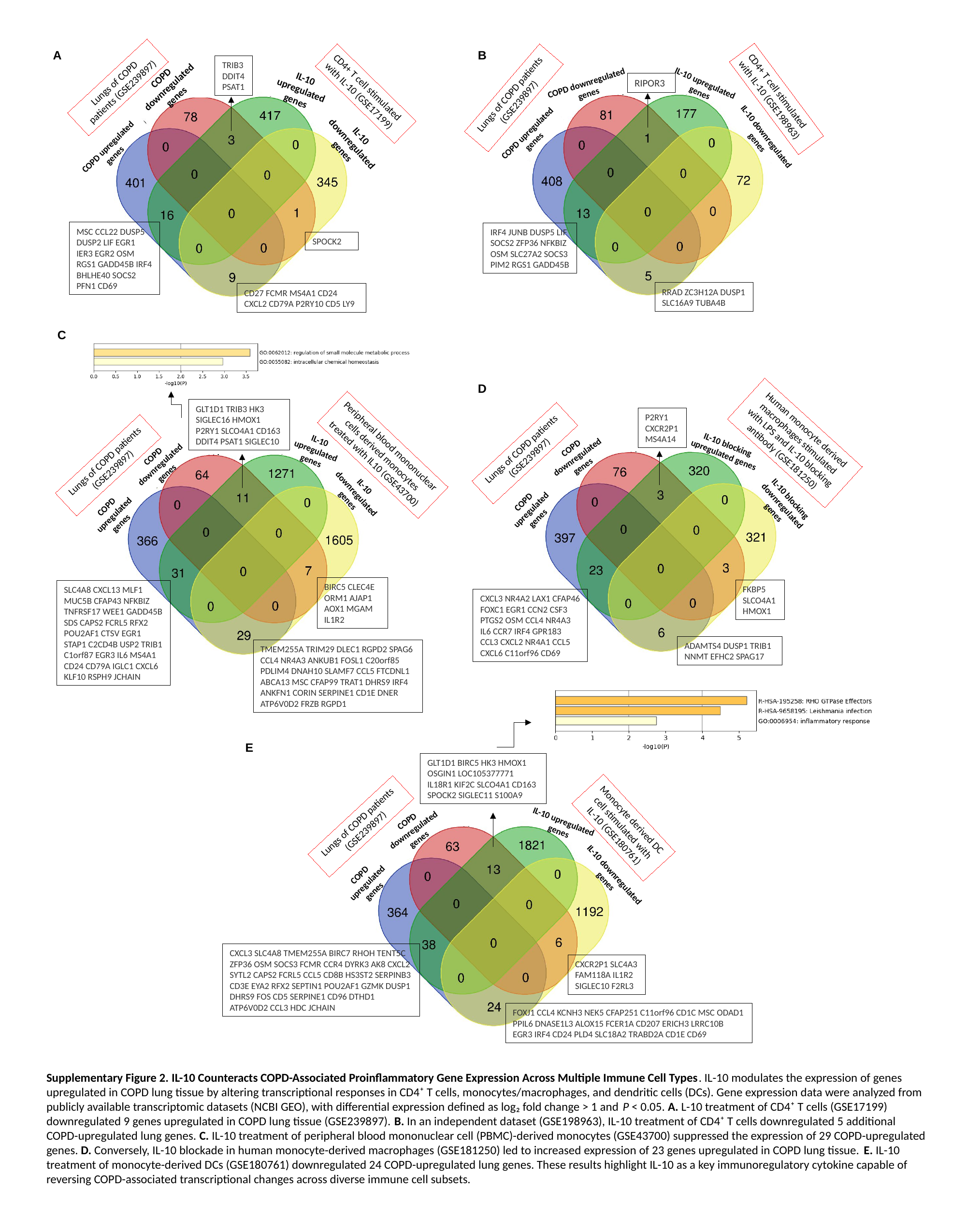

RIPOR3
CD4+ T cell stimulated with IL-10 (GSE198963)
IRF4 JUNB DUSP5 LIF SOCS2 ZFP36 NFKBIZ OSM SLC27A2 SOCS3 PIM2 RGS1 GADD45B
RRAD ZC3H12A DUSP1 SLC16A9 TUBA4B
COPD downregulated genes
IL-10 upregulated genes
Lungs of COPD patients (GSE239897)
COPD upregulated genes
IL-10 downregulated genes
A
B
TRIB3 DDIT4 PSAT1
CD4+ T cell stimulated with IL-10 (GSE17199)
MSC CCL22 DUSP5 DUSP2 LIF EGR1 IER3 EGR2 OSM RGS1 GADD45B IRF4 BHLHE40 SOCS2 PFN1 CD69
SPOCK2
CD27 FCMR MS4A1 CD24 CXCL2 CD79A P2RY10 CD5 LY9
COPD downregulated genes
IL-10 upregulated genes
Lungs of COPD patients (GSE239897)
IL-10 downregulated genes
COPD upregulated genes
C
GLT1D1 TRIB3 HK3 SIGLEC16 HMOX1 P2RY1 SLCO4A1 CD163 DDIT4 PSAT1 SIGLEC10
IL-10 upregulated genes
Peripheral blood mononuclear cells derived monocytes treated with IL10 (GSE43700)
COPD downregulated genes
Lungs of COPD patients (GSE239897)
IL-10 downregulated genes
COPD upregulated genes
BIRC5 CLEC4E ORM1 AJAP1 AOX1 MGAM IL1R2
SLC4A8 CXCL13 MLF1 MUC5B CFAP43 NFKBIZ TNFRSF17 WEE1 GADD45B SDS CAPS2 FCRL5 RFX2 POU2AF1 CTSV EGR1 STAP1 C2CD4B USP2 TRIB1 C1orf87 EGR3 IL6 MS4A1 CD24 CD79A IGLC1 CXCL6 KLF10 RSPH9 JCHAIN
TMEM255A TRIM29 DLEC1 RGPD2 SPAG6 CCL4 NR4A3 ANKUB1 FOSL1 C20orf85 PDLIM4 DNAH10 SLAMF7 CCL5 FTCDNL1 ABCA13 MSC CFAP99 TRAT1 DHRS9 IRF4 ANKFN1 CORIN SERPINE1 CD1E DNER ATP6V0D2 FRZB RGPD1
D
P2RY1 CXCR2P1 MS4A14
IL-10 blocking upregulated genes
FKBP5 SLCO4A1 HMOX1
CXCL3 NR4A2 LAX1 CFAP46 FOXC1 EGR1 CCN2 CSF3 PTGS2 OSM CCL4 NR4A3 IL6 CCR7 IRF4 GPR183 CCL3 CXCL2 NR4A1 CCL5 CXCL6 C11orf96 CD69
ADAMTS4 DUSP1 TRIB1 NNMT EFHC2 SPAG17
COPD downregulated genes
Lungs of COPD patients (GSE239897)
IL-10 blocking downregulated genes
COPD upregulated genes
Human monocyte derived macrophages stimulated with LPS and IL-10 blocking antibody (GSE181250)
E
GLT1D1 BIRC5 HK3 HMOX1 OSGIN1 LOC105377771 IL18R1 KIF2C SLCO4A1 CD163 SPOCK2 SIGLEC11 S100A9
Monocyte derived DC cell stimulated with IL-10 (GSE180761)
CXCL3 SLC4A8 TMEM255A BIRC7 RHOH TENT5C ZFP36 OSM SOCS3 FCMR CCR4 DYRK3 AK8 CXCL2 SYTL2 CAPS2 FCRL5 CCL5 CD8B HS3ST2 SERPINB3 CD3E EYA2 RFX2 SEPTIN1 POU2AF1 GZMK DUSP1 DHRS9 FOS CD5 SERPINE1 CD96 DTHD1 ATP6V0D2 CCL3 HDC JCHAIN
CXCR2P1 SLC4A3 FAM118A IL1R2 SIGLEC10 F2RL3
FOXJ1 CCL4 KCNH3 NEK5 CFAP251 C11orf96 CD1C MSC ODAD1 PPIL6 DNASE1L3 ALOX15 FCER1A CD207 ERICH3 LRRC10B EGR3 IRF4 CD24 PLD4 SLC18A2 TRABD2A CD1E CD69
COPD downregulated genes
Lungs of COPD patients (GSE239897)
IL-10 upregulated genes
COPD upregulated genes
IL-10 downregulated genes
Supplementary Figure 2. IL-10 Counteracts COPD-Associated Proinflammatory Gene Expression Across Multiple Immune Cell Types. IL-10 modulates the expression of genes upregulated in COPD lung tissue by altering transcriptional responses in CD4⁺ T cells, monocytes/macrophages, and dendritic cells (DCs). Gene expression data were analyzed from publicly available transcriptomic datasets (NCBI GEO), with differential expression defined as log₂ fold change > 1 and P < 0.05. A. L-10 treatment of CD4⁺ T cells (GSE17199) downregulated 9 genes upregulated in COPD lung tissue (GSE239897). B. In an independent dataset (GSE198963), IL-10 treatment of CD4⁺ T cells downregulated 5 additional COPD-upregulated lung genes. C. IL-10 treatment of peripheral blood mononuclear cell (PBMC)-derived monocytes (GSE43700) suppressed the expression of 29 COPD-upregulated genes. D. Conversely, IL-10 blockade in human monocyte-derived macrophages (GSE181250) led to increased expression of 23 genes upregulated in COPD lung tissue. E. IL-10 treatment of monocyte-derived DCs (GSE180761) downregulated 24 COPD-upregulated lung genes. These results highlight IL-10 as a key immunoregulatory cytokine capable of reversing COPD-associated transcriptional changes across diverse immune cell subsets.

## Slide 7
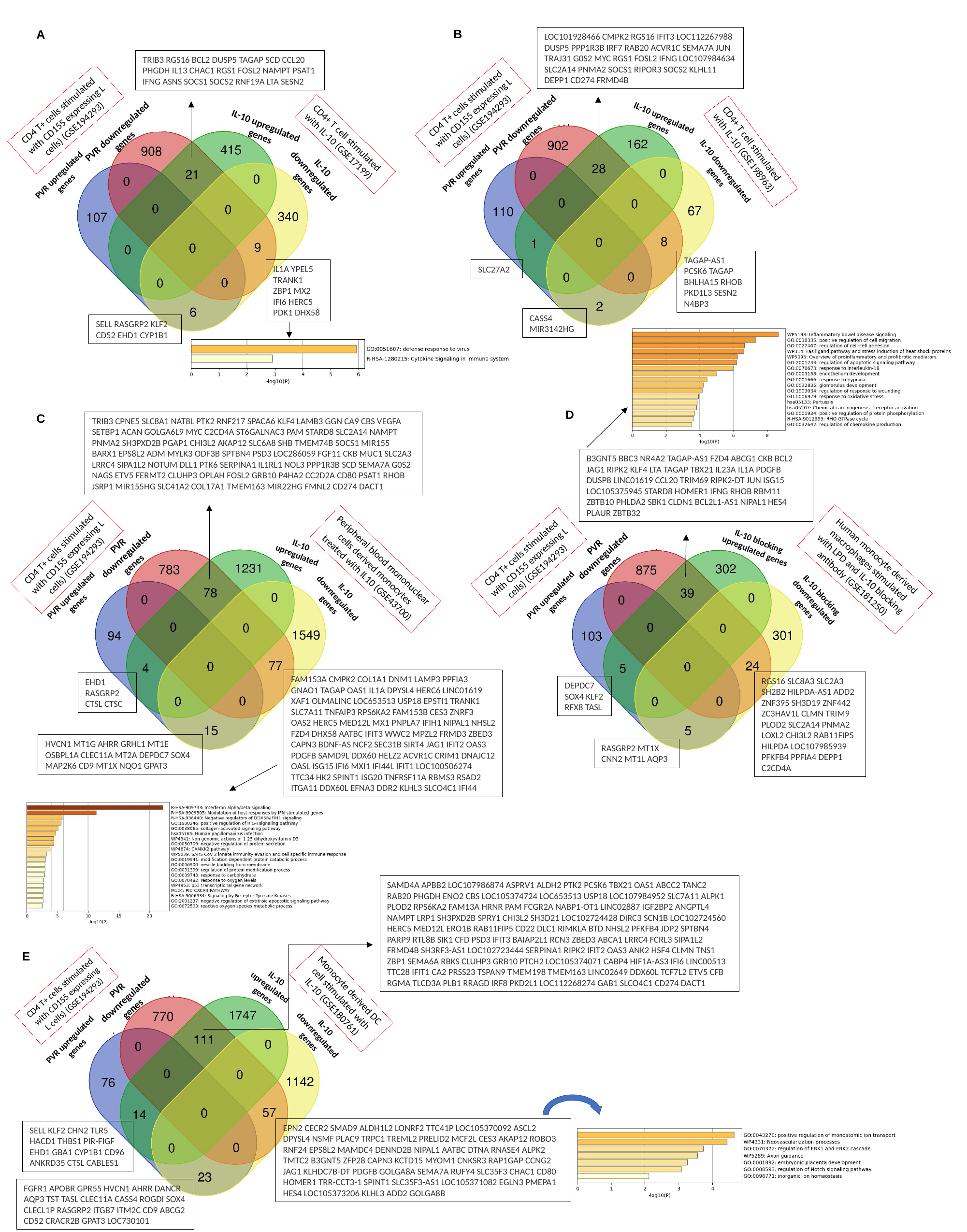

B
A
LOC101928466 CMPK2 RGS16 IFIT3 LOC112267988 DUSP5 PPP1R3B IRF7 RAB20 ACVR1C SEMA7A JUN TRAJ31 G0S2 MYC RGS1 FOSL2 IFNG LOC107984634 SLC2A14 PNMA2 SOCS1 RIPOR3 SOCS2 KLHL11 DEPP1 CD274 FRMD4B
CD4 T+ cells stimulated with CD155 expressing L cells) (GSE194293)
IL-10 upregulated genes
PVR downregulated genes
CD4+ T cell stimulated with IL-10 (GSE198963)
PVR upregulated genes
IL-10 downregulated genes
TAGAP-AS1 PCSK6 TAGAP BHLHA15 RHOB PKD1L3 SESN2 N4BP3
SLC27A2
CASS4 MIR3142HG
TRIB3 RGS16 BCL2 DUSP5 TAGAP SCD CCL20 PHGDH IL13 CHAC1 RGS1 FOSL2 NAMPT PSAT1 IFNG ASNS SOCS1 SOCS2 RNF19A LTA SESN2
CD4 T+ cells stimulated with CD155 expressing L cells) (GSE194293)
IL-10 upregulated genes
PVR downregulated genes
CD4+ T cell stimulated with IL-10 (GSE17199)
IL-10 downregulated genes
PVR upregulated genes
IL1A YPEL5 TRANK1 ZBP1 MX2 IFI6 HERC5 PDK1 DHX58
SELL RASGRP2 KLF2 CD52 EHD1 CYP1B1
D
C
TRIB3 CPNE5 SLC8A1 NAT8L PTK2 RNF217 SPACA6 KLF4 LAMB3 GGN CA9 CBS VEGFA SETBP1 ACAN GOLGA6L9 MYC C2CD4A ST6GALNAC3 PAM STARD8 SLC2A14 NAMPT PNMA2 SH3PXD2B PGAP1 CHI3L2 AKAP12 SLC6A8 SHB TMEM74B SOCS1 MIR155 BARX1 EPS8L2 ADM MYLK3 ODF3B SPTBN4 PSD3 LOC286059 FGF11 CKB MUC1 SLC2A3 LRRC4 SIPA1L2 NOTUM DLL1 PTK6 SERPINA1 IL1RL1 NOL3 PPP1R3B SCD SEMA7A G0S2 NAGS ETV5 FERMT2 CLUHP3 OPLAH FOSL2 GRB10 P4HA2 CC2D2A CD80 PSAT1 RHOB JSRP1 MIR155HG SLC41A2 COL17A1 TMEM163 MIR22HG FMNL2 CD274 DACT1
FAM153A CMPK2 COL1A1 DNM1 LAMP3 PPFIA3 GNAO1 TAGAP OAS1 IL1A DPYSL4 HERC6 LINC01619 XAF1 OLMALINC LOC653513 USP18 EPSTI1 TRANK1 SLC7A11 TNFAIP3 RPS6KA2 FAM153B CES3 ZNRF3 OAS2 HERC5 MED12L MX1 PNPLA7 IFIH1 NIPAL1 NHSL2 FZD4 DHX58 AATBC IFIT3 WWC2 MPZL2 FRMD3 ZBED3 CAPN3 BDNF-AS NCF2 SEC31B SIRT4 JAG1 IFIT2 OAS3 PDGFB SAMD9L DDX60 HELZ2 ACVR1C CRIM1 DNAJC12 OASL ISG15 IFI6 MXI1 IFI44L IFIT1 LOC100506274 TTC34 HK2 SPINT1 ISG20 TNFRSF11A RBMS3 RSAD2 ITGA11 DDX60L EFNA3 DDR2 KLHL3 SLCO4C1 IFI44
EHD1 RASGRP2 CTSL CTSC
HVCN1 MT1G AHRR GRHL1 MT1E OSBPL1A CLEC11A MT2A DEPDC7 SOX4 MAP2K6 CD9 MT1X NQO1 GPAT3
PVR downregulated genes
IL-10 upregulated genes
CD4 T+ cells stimulated with CD155 expressing L cells) (GSE194293)
Peripheral blood mononuclear cells derived monocytes treated with IL10 (GSE43700)
IL-10 downregulated genes
PVR upregulated genes
B3GNT5 BBC3 NR4A2 TAGAP-AS1 FZD4 ABCG1 CKB BCL2 JAG1 RIPK2 KLF4 LTA TAGAP TBX21 IL23A IL1A PDGFB DUSP8 LINC01619 CCL20 TRIM69 RIPK2-DT JUN ISG15 LOC105375945 STARD8 HOMER1 IFNG RHOB RBM11 ZBTB10 PHLDA2 SBK1 CLDN1 BCL2L1-AS1 NIPAL1 HES4 PLAUR ZBTB32
IL-10 blocking upregulated genes
PVR downregulated genes
CD4 T+ cells stimulated with CD155 expressing L cells) (GSE194293)
Human monocyte derived macrophages stimulated with LPD and IL-10 blocking antibody (GSE181250)
IL-10 blocking downregulated genes
PVR upregulated genes
RGS16 SLC8A3 SLC2A3 SH2B2 HILPDA-AS1 ADD2 ZNF395 SH3D19 ZNF442 ZC3HAV1L CLMN TRIM9 PLOD2 SLC2A14 PNMA2 LOXL2 CHI3L2 RAB11FIP5 HILPDA LOC107985939 PFKFB4 PPFIA4 DEPP1 C2CD4A
DEPDC7 SOX4 KLF2 RFX8 TASL
RASGRP2 MT1X CNN2 MT1L AQP3
SAMD4A APBB2 LOC107986874 ASPRV1 ALDH2 PTK2 PCSK6 TBX21 OAS1 ABCC2 TANC2 RAB20 PHGDH ENO2 CBS LOC105374724 LOC653513 USP18 LOC107984952 SLC7A11 ALPK1 PLOD2 RPS6KA2 FAM13A HRNR PAM FCGR2A NABP1-OT1 LINC02887 IGF2BP2 ANGPTL4 NAMPT LRP1 SH3PXD2B SPRY1 CHI3L2 SH3D21 LOC102724428 DIRC3 SCN1B LOC102724560 HERC5 MED12L ERO1B RAB11FIP5 CD22 DLC1 RIMKLA BTD NHSL2 PFKFB4 JDP2 SPTBN4 PARP9 RTL8B SIK1 CFD PSD3 IFIT3 BAIAP2L1 RCN3 ZBED3 ABCA1 LRRC4 FCRL3 SIPA1L2 FRMD4B SH3RF3-AS1 LOC102723444 SERPINA1 RIPK2 IFIT2 OAS3 ANK2 HSF4 CLMN TNS1 ZBP1 SEMA6A RBKS CLUHP3 GRB10 PTCH2 LOC105374071 CABP4 HIF1A-AS3 IFI6 LINC00513 TTC28 IFIT1 CA2 PRSS23 TSPAN9 TMEM198 TMEM163 LINC02649 DDX60L TCF7L2 ETV5 CFB RGMA TLCD3A PLB1 RRAGD IRF8 PKD2L1 LOC112268274 GAB1 SLCO4C1 CD274 DACT1
IL-10 upregulated genes
CD4 T+ cells stimulated with CD155 expressing L cells) (GSE194293)
Monocyte derived DC cell stimulated with IL-10 (GSE180761)
PVR downregulated genes
IL-10 downregulated genes
PVR upregulated genes
EPN2 CECR2 SMAD9 ALDH1L2 LONRF2 TTC41P LOC105370092 ASCL2 DPYSL4 NSMF PLAC9 TRPC1 TREML2 PRELID2 MCF2L CES3 AKAP12 ROBO3 RNF24 EPS8L2 MAMDC4 DENND2B NIPAL1 AATBC DTNA RNASE4 ALPK2 TMTC2 B3GNT5 ZFP28 CAPN3 KCTD15 MYOM1 CNKSR3 RAP1GAP CCNG2 JAG1 KLHDC7B-DT PDGFB GOLGA8A SEMA7A RUFY4 SLC35F3 CHAC1 CD80 HOMER1 TRR-CCT3-1 SPINT1 SLC35F3-AS1 LOC105371082 EGLN3 PMEPA1 HES4 LOC105373206 KLHL3 ADD2 GOLGA8B
SELL KLF2 CHN2 TLR5 HACD1 THBS1 PIR-FIGF EHD1 GBA1 CYP1B1 CD96 ANKRD35 CTSL CABLES1
FGFR1 APOBR GPR55 HVCN1 AHRR DANCR AQP3 TST TASL CLEC11A CASS4 ROGDI SOX4 CLECL1P RASGRP2 ITGB7 ITM2C CD9 ABCG2 CD52 CRACR2B GPAT3 LOC730101
E

## Slide 8
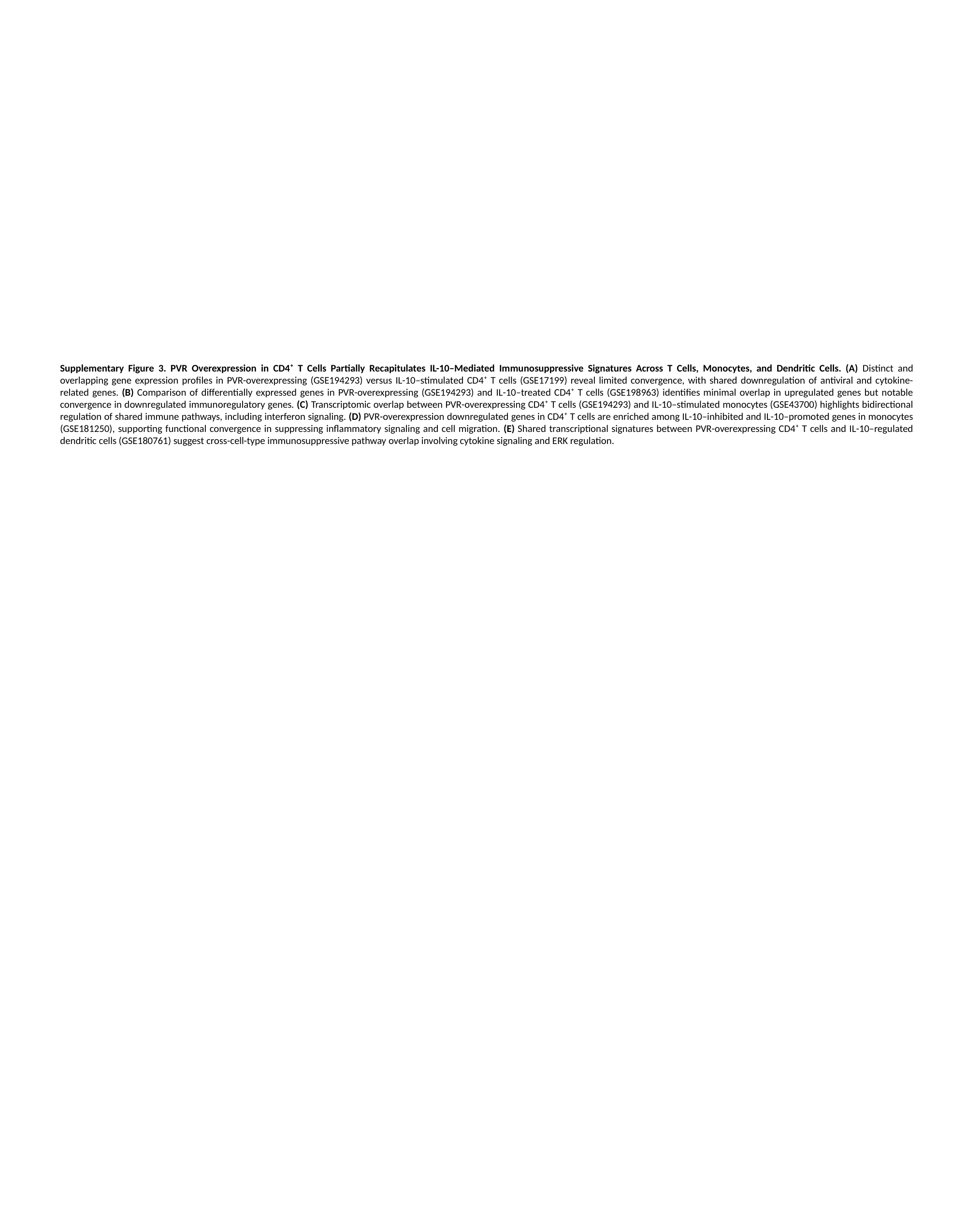

Supplementary Figure 3. PVR Overexpression in CD4⁺ T Cells Partially Recapitulates IL-10–Mediated Immunosuppressive Signatures Across T Cells, Monocytes, and Dendritic Cells. (A) Distinct and overlapping gene expression profiles in PVR-overexpressing (GSE194293) versus IL-10–stimulated CD4⁺ T cells (GSE17199) reveal limited convergence, with shared downregulation of antiviral and cytokine-related genes. (B) Comparison of differentially expressed genes in PVR-overexpressing (GSE194293) and IL-10–treated CD4⁺ T cells (GSE198963) identifies minimal overlap in upregulated genes but notable convergence in downregulated immunoregulatory genes. (C) Transcriptomic overlap between PVR-overexpressing CD4⁺ T cells (GSE194293) and IL-10–stimulated monocytes (GSE43700) highlights bidirectional regulation of shared immune pathways, including interferon signaling. (D) PVR-overexpression downregulated genes in CD4⁺ T cells are enriched among IL-10–inhibited and IL-10–promoted genes in monocytes (GSE181250), supporting functional convergence in suppressing inflammatory signaling and cell migration. (E) Shared transcriptional signatures between PVR-overexpressing CD4⁺ T cells and IL-10–regulated dendritic cells (GSE180761) suggest cross-cell-type immunosuppressive pathway overlap involving cytokine signaling and ERK regulation.

## Slide 9
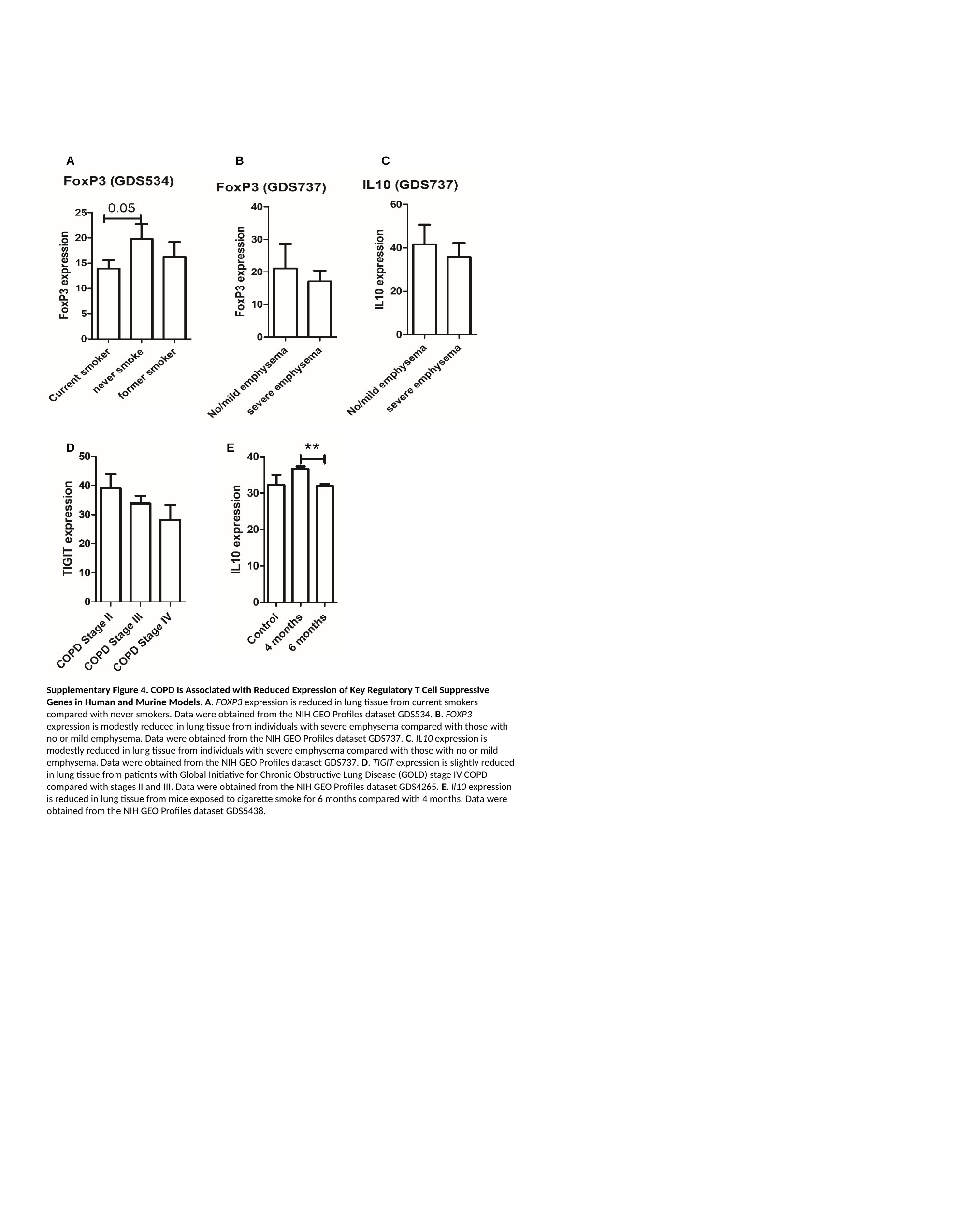

A
B
C
E
D
Supplementary Figure 4. COPD Is Associated with Reduced Expression of Key Regulatory T Cell Suppressive Genes in Human and Murine Models. A. FOXP3 expression is reduced in lung tissue from current smokers compared with never smokers. Data were obtained from the NIH GEO Profiles dataset GDS534. B. FOXP3 expression is modestly reduced in lung tissue from individuals with severe emphysema compared with those with no or mild emphysema. Data were obtained from the NIH GEO Profiles dataset GDS737. C. IL10 expression is modestly reduced in lung tissue from individuals with severe emphysema compared with those with no or mild emphysema. Data were obtained from the NIH GEO Profiles dataset GDS737. D. TIGIT expression is slightly reduced in lung tissue from patients with Global Initiative for Chronic Obstructive Lung Disease (GOLD) stage IV COPD compared with stages II and III. Data were obtained from the NIH GEO Profiles dataset GDS4265. E. Il10 expression is reduced in lung tissue from mice exposed to cigarette smoke for 6 months compared with 4 months. Data were obtained from the NIH GEO Profiles dataset GDS5438.
